# Childhood maltreatment and mental health problems: A systematic review and meta-analysis of quasi-experimental studies

**DOI:** 10.1176/appi.ajp.20220174

**Published:** 2023-01-11

**Authors:** Jessie R. Baldwin, Biyao Wang, Lucy Karwatowska, Tabea Schoeler, Anna Tsaligopoulou, Marcus R. Munafò, Jean-Baptiste Pingault

**Affiliations:** 1 Department of Clinical, Educational and Health Psychology, Division of Psychology and Language Sciences, University College London, London, UK; 2 Social, Genetic and Developmental Psychiatry Centre, Institute of Psychiatry, Psychology and Neuroscience, King’s College London, London, UK; 3 Great Ormond Street Institute of Child Health, University College London, London, UK; 4 Department of Computational Biology, University of Lausanne, Lausanne, Switzerland; 5 Child Study Center, Yale University School of Medicine, New Haven, CT, USA; 6 MRC Integrative Epidemiology Unit at the University of Bristol, Bristol Medical School, University of Bristol, Bristol, UK; 7 School of Psychological Science, University of Bristol, Bristol, UK; 8 NIHR Biomedical Research Centre, University Hospitals Bristol NHS Foundation Trust and University of Bristol, Bristol, UK

## Abstract

**Objective:**

Childhood maltreatment is associated with mental health problems, but the extent to which this relationship is causal remains unclear. To strengthen causal inference, we conducted a systematic review and meta-analysis of quasi-experimental studies examining the relationship between childhood maltreatment and mental health problems.

**Methods:**

We searched PubMed, PsycINFO, and Embase for peer-reviewed, English language articles from inception until January 1, 2022. Studies were included if they examined the association between childhood maltreatment and mental health problems using a quasi-experimental method (e.g., twin/sibling differences design, Children of Twins design, adoption design, fixed-effects design, random-intercept cross-lagged panel model, natural experiment, propensity score matching, or inverse probability weighting).

**Results:**

We identified 34 quasi-experimental studies, including 54,646 independent participants. Before quasi-experimental adjustment for confounding, childhood maltreatment was moderately associated with mental health problems (Cohen’s d=0.56, 95% CI=0.41-0.71). Following quasi-experimental adjustment, a small association between childhood maltreatment and mental health problems remained (Cohen’s d=0.31, 95% CI=0.24-0.37). This adjusted association between child maltreatment and mental health was consistent across different quasi-experimental methods, and generalised across different psychiatric disorders.

**Conclusion:**

These findings are consistent with a small, causal contribution of childhood maltreatment to mental health problems. Furthermore, the findings suggest that part of the overall risk of mental health problems in individuals exposed to maltreatment is due to wider genetic and environmental risk factors. Therefore, preventing childhood maltreatment and addressing wider psychiatric risk factors in individuals exposed to maltreatment could help to prevent psychopathology.

## Introduction

Childhood maltreatment is a well-established risk factor for mental health problems. For example, systematic reviews and meta-analyses consistently show associations between childhood maltreatment and a range of psychiatric disorders, such as depression (1), anxiety (2), psychosis (3), suicidality (4), non-suicidal self-injury (5), attention-deficit hyperactivity disorder (ADHD) (6), conduct disorder (7), and substance abuse (8). Understanding the causal nature of these associations is critical for informing preventative interventions.

Maltreated children might be more likely to develop mental health problems by virtue of their exposure to abuse and neglect. Alternatively, their risk might be higher because they have other genetic and environmental risk factors for mental health problems, which confound previously observed associations. For example, evidence suggests that maltreated children are likely to have family histories of mental illness (9) and may have higher polygenic scores for psychiatric disorders (e.g., schizophrenia and depression) (10, 11). Maltreated children are also likely to experience other environmental risks for mental health problems, such as socioeconomic disadvantage (9) and bullying victimization (12). However, these co-occurring risk factors have not been not fully accounted for in the majority of previous research on maltreatment and mental health, which used classical epidemiological methods (e.g., multiple regression) with limited ability to account for genetic influences or other unmeasured confounders.

Quasi-experimental methods offer a solution to help disentangle the causal effects of child maltreatment on mental health from confounding (13). Four broad categories of quasi-experimental methods have been used to examine the relationship between maltreatment and mental health. First, *family-based designs* (e.g., twin or sibling differences, Children of Twins [CoT], and adoption designs; 14) capitalize on varying genetic and environmental relationships between family members to examine the effects of maltreatment independent of familial confounding. Second, *panel data designs* (e.g., fixed-effects methods, random intercept cross-lagged models) leverage longitudinal data to test whether within-individual changes in maltreatment exposure predict changes in mental health, independent of stable individual factors. Third, *natural experiments* examine the effects of maltreatment that is not related to family or individual risk factors, but rather occurs due to wider social or political processes. For example, the English and Romanian Adoptees (ERA) Study (15) examined the mental health consequences of institutional neglect that occurred during the Romanian Ceauşescu regime, in which widespread poverty and bans on contraception led to thousands of babies being abandoned in orphanages. Fourth, *propensity score methods* (e.g., propensity score matching; inverse probability weighting) aim to mimic a randomized experiment by statistically removing confounding by (measured) pre-existing differences between maltreated and non-maltreated individuals. Although propensity score methods do not directly account for unmeasured confounding, they have been found to be more effective than multiple regression in reducing confounding (16).

While a number of individual studies have applied these quasi-experimental methods to strengthen causal inference about the relationship between maltreatment and mental health, there has been no systematic summary of this evidence. Such a systematic evaluation is important to (1) estimate the pooled magnitude of a potentially causal relationship between maltreatment and mental health, (2) examine whether findings triangulate across quasi-experimental methods with different assumptions and sources of bias, and (3) examine whether findings differ according to key moderators, such as the type of mental health outcome, form of maltreatment, or assessment method. To address this research gap, we conducted the first systematic review and meta-analysis of quasi-experimental studies on childhood maltreatment and mental health problems.

## Methods

We performed a systematic review and meta-analysis following a pre-registered protocol on Prospero (CRD42020187520), adhering to PRISMA and MOOSE reporting guidelines (Tables S1-2).

### Search strategy

We searched Embase, PsycINFO, and MEDLINE for peer-reviewed studies written in English and published from database inception until January 1, 2022. Search terms are reported in Methods S1 of the Supplement. Two investigators (J.R.B and B.W) independently screened titles and abstracts of all articles retrieved from the search, and reviewed the full texts of potentially eligible studies. Inter-rater agreement was high (88%) and remaining disagreement was resolved through discussion with a third investigator (J.B.P).

### Study selection

We included studies that:

1. Included measures of childhood maltreatment in humans, defined as any of the following experiences before age 18: physical abuse, sexual abuse, emotional abuse, physical neglect, emotional neglect, institutional neglect/deprivation, harsh physical discipline/corporal punishment, or broader measures of victimization/adverse childhood experiences (ACEs) that included the above forms of maltreatment. As such, the primary focus of this meta-analysis was on maltreatment rather than ACEs more generally, as in (as in 17).
2. Examined associations with mental health outcomes, defined as diagnoses or symptoms of internalizing, externalizing, neurodevelopmental, or thought disorders, or general psychopathology, assessed concurrent to or after the observational period for maltreatment.
3. Used a quasi-experimental design (see Tables S3-S4 for an overview of included and excluded designs).

### Data extraction

Two investigators (J.R.B and either L.K or A.T) independently extracted data from all relevant studies (see Methods S2 of the Supplement for details). For all studies, we extracted adjusted effect sizes from quasi-experimental methods. Where multiple quasi-experimental estimates were presented (e.g., effect sizes from dizygotic [DZ] and monozygotic [MZ] twin difference analyses), we selected the most stringent estimate (e.g., MZ twin difference estimates). For comparison, we also extracted unadjusted effect sizes, where this was reported or appropriate (e.g., it was not possible for natural experiment studies). We coded study quality using an adapted version of the Newcastle-Ottowa scale (see Methods S3 of the Supplement for details).

### Effect size conversion

We converted the individual study effect sizes to Cohen’s d values, reflecting the standardised mean difference in mental health problems between maltreated and non-maltreated individuals. Methods and formula used to convert effect sizes are reported in Methods S4 and Table S5. We excluded a subset of effect sizes from an included study (18) presented as negative binomial regression coefficients, because they could not be converted to Cohen’s d without additional unavailable information. We were also unable to convert unstandardised coefficients from a Mendelian randomisation study (19) to Cohen’s d because the nature of the underlying data meant that the conversion was not appropriate. Excluded effect sizes are reported in Table S6.

### Statistical analysis

We conducted all analyses in R (version 4.1.1) using the *metafor* and *dmetar* packages (20, 21). The script and dataset are available at https://github.com/jr-baldwin/maltreatment_MH_meta. We performed multi-level random-effects meta-analysis models (22), which account for dependencies among multiple effect sizes per study and/or cohort (e.g., where multiple mental health outcomes or maltreatment subtypes were examined). We specified four levels of variance in effect sizes: (1) random-sampling variance, (2) within-study (i.e., within-article) variance, (3) between-study variance, and (4) between samples variance, and applied robust standard errors to the main meta-analytic models. We first examined the meta-analytic association between maltreatment and mental health in quasi-experimental adjusted estimates, and then examined the meta-analytic association in studies reporting unadjusted effect sizes, for comparison. We estimated heterogeneity through the I^2^ statistic, which reflects the proportion of the observed variance that is due to variation in true effect sizes if sampling error was eliminated.

We conducted sensitivity analyses testing for publication bias, undue influence of individual studies, cohorts, or effect sizes, and robustness of the findings to the definition of maltreatment used. To test for publication bias, we used an Egger’s test for multi-level meta-analysis models (23), which tests whether the meta-analytic effect size is moderated by the study variance. We also inspected funnel plots and conducted a p-curve analysis (24) based on an average effect size per study to test whether statistically significant findings were likely to reflect selective reporting and/or p-hacking. To test for undue influence of individual studies, cohorts, or effect sizes, we conducted three leave-one-out analyses testing changes in the estimate across permutations omitting in turn each study, cohort, and effect size, respectively. To test the robustness of the findings to the definition of maltreatment, we re-ran the meta-analysis including only studies assessing child maltreatment in the home, and excluded studies that focused on institutional neglect or assessed maltreatment along with other forms of victimization or ACEs.

Lastly, we used meta-regression to test whether the quasi-experimental association between childhood maltreatment and mental health was moderated by a-priori selected factors, including type of quasi-experimental method, mental health outcome, maltreatment subtype, measurement characteristics, sample characteristics, and study quality. Sensitivity analyses were performed to test whether the moderation results differed after excluding a potentially influential cohort.

## Results

### Search results

The study selection procedure is summarized in Figure S1. We identified 35 quasi-experimental studies on the association between child maltreatment and mental health, of which 34 had available data that could be meta-analysed (15, 18, 25–56) (see Table S7 for study details). These studies were based on 29 distinct cohorts, comprising 54,646 participants in adjusted analyses (56.72% female, with a mean age of 28.2 years at mental health assessment). From these studies, we obtained 156 effect sizes for the association between child maltreatment and mental health based on adjusted analyses and 103 effect sizes based on unadjusted analyses.

### Quasi-experimental evidence on the association between child maltreatment and mental health

**Figure 1:**
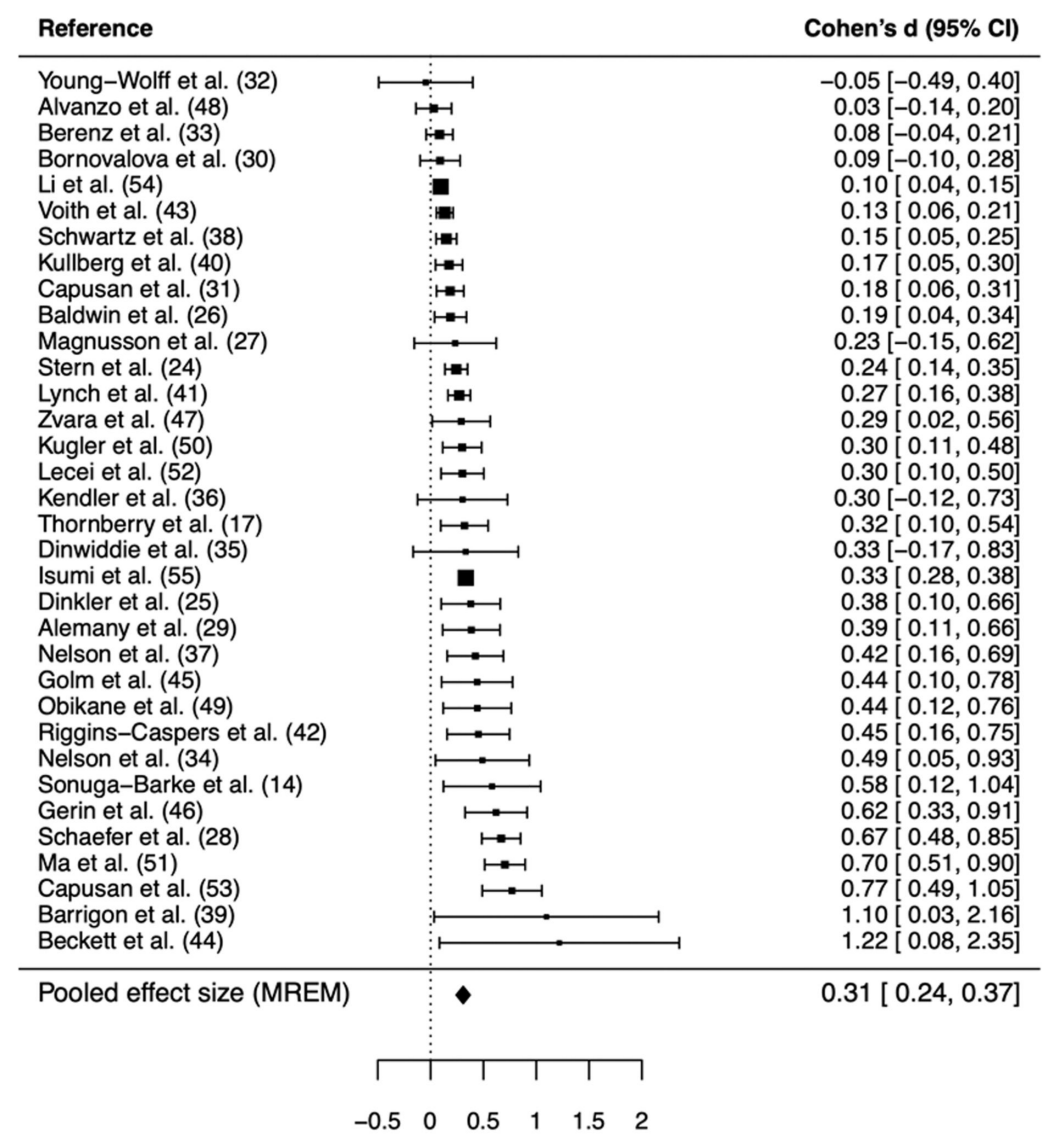
Forest plot depicting the study-average effects of child maltreatment on mental health from quasi-experimental studies. Note. MREM = multi-level random-effects meta-analysis model. For clarity of presentation, the forest plot shows 1 effect size per study (reflecting the average of all individual effect sizes obtained from each study), rather than all 150 effect sizes used to derive the pooled MREM estimate. The average effect size per study and its variance was calculated using the MAd package,(87) assuming a correlation of 0.6 between multiple within-study effect sizes. The 156 effect sizes are presented in Table S9 of the Supplement. *I*^2^ for the MREM was 76.27, indicating that 76% of variation between effect sizes would remain if sampling error was eliminated.

A multilevel random-effects meta-analysis model showed a small association between childhood maltreatment and mental health problems in quasi-experimental studies (Cohen’s d=0.31, 95% CI=0.24-0.37, *I*^2^=76.27; Figure 1). This meta-analytic association between child maltreatment and mental health from quasi-experimental studies was 45% smaller than that obtained in unadjusted analyses (k=20; Cohen’s d=0.56, 95% CI=0.41-0.71, *I*^2^=97.29 [Figure S2]; p-value for difference=0.004), suggesting that the unadjusted association is inflated by confounding. This difference in effect size was broadly consistent when the quasi-experimental adjusted meta-analysis was restricted to studies reporting both unadjusted and adjusted effect sizes (k=20; Cohen’s d=0.26, 95% CI=0.17-0.35).

### Sensitivity analyses

An Egger’s test suggested evidence of small-study bias (Q_moderation=8.52, p-value=0.004; see Figure S3A for funnel plot). We investigated the cause of this potential publication bias by performing leave-one-out analyses and found that it was due to the inclusion of data from one cohort, the ERA Study (15, 45, 46), which comprised a comparatively small sample (N=90-148) with large effect sizes linked to severe institutional neglect (Q_moderation after excluding ERA=2.92, p-value=0.09; see Figure S3B for funnel plot). Where relevant, we therefore conducted later moderation analyses both with and without effect sizes from the ERA cohort, to ensure that results were not biased. The p-curve analysis (focusing on one averaged effect size per study) provided evidential value that a true effect was present (Figure S4).

Leave-one-out analyses showed that the overall meta-analytic estimate was not unduly influenced by individual studies, cohorts, or effect sizes. The meta-analytic effect size ranged between Cohen’s d=0.29 to 0.32 (with overlapping confidence intervals) after omitting in turn each of the 29 cohorts (including ERA), 34 studies, and 156 effect sizes (Figures S5-6). After excluding studies which assessed maltreatment as part of broader measures of victimization (k=3) (25, 27, 29) or ACEs (k=5) (30, 34, 39, 44, 49), or focused on institutional neglect (k=3) (15, 45, 46), the meta-analytic effect size was also similar to the original estimate (Cohen’s d=0.34, 95% CI=0.26-0.42).

### Moderators of the association between child maltreatment and mental health

#### Type of quasi-experimental method

**Figure 2:**
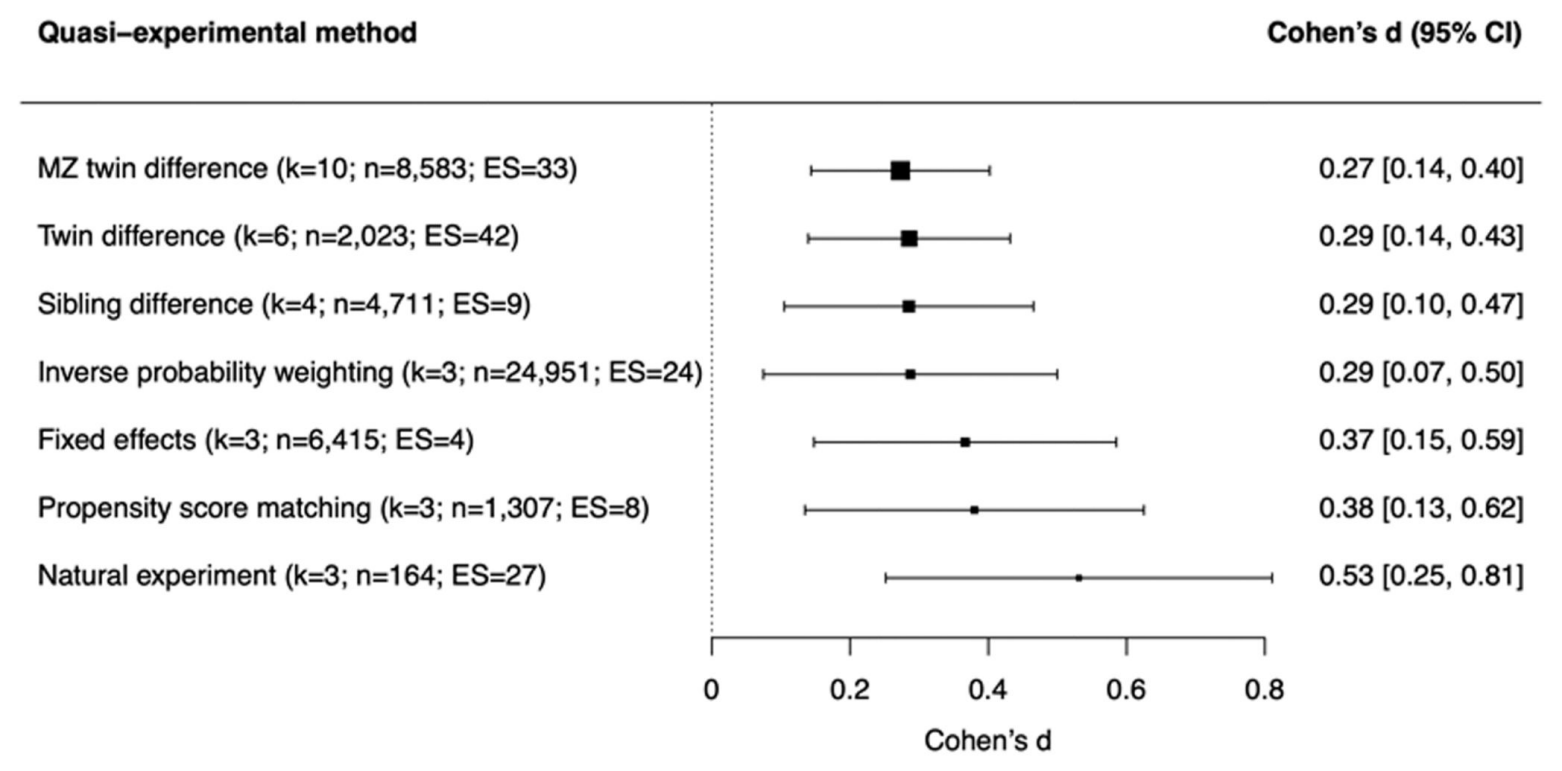
Meta-analytic associations between child maltreatment and mental health problems across different quasi-experimental methods. Note. k=number of studies (i.e., papers); n=number of participants across studies; ES=number of effect sizes. “MZ twin difference” refers to twin difference designs including only monozygotic twins, while “twin difference” refers to twin difference designs that include both monozygotic and dizygotic twins. Quasi-experimental methods used by only a single study in the meta-analysis (namely the adoption design (43), Children of Twins design (42), and random-intercept cross-lagged panel model (55)) were not included in the moderator analysis.

As shown in Figure 2, the association between child maltreatment and mental health was present across different quasi-experimental designs (twin differences, sibling differences, fixed-effects, and natural experiment) and analytic approaches (propensity score matching and inverse probability weighting) and was not significantly moderated by the type of quasi-experimental method used (Q_moderation=3.43; p-value=0.75).

#### Type of mental health outcome

**Figure 3:**
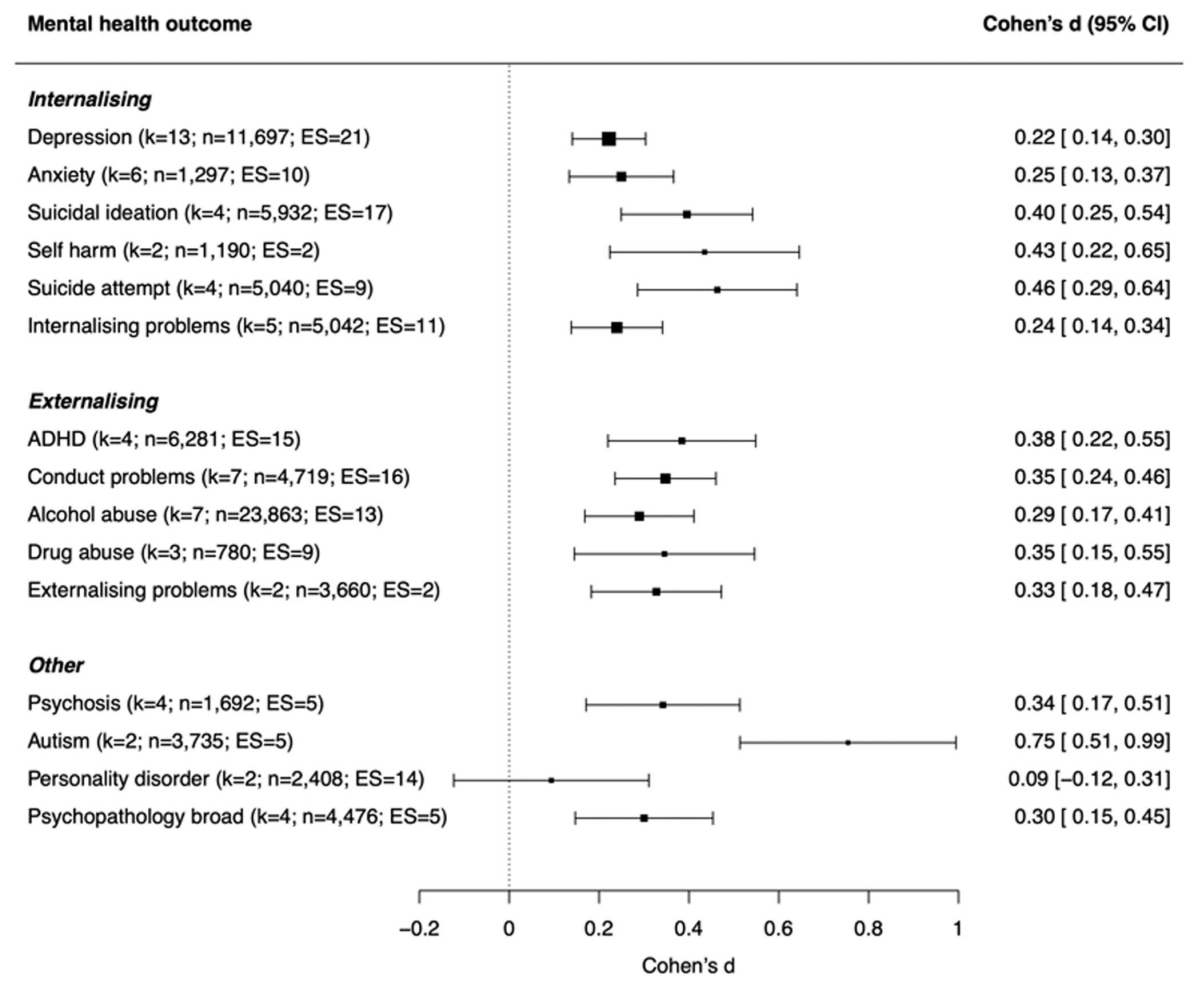
Meta-analytic associations between child maltreatment and different mental health problems *Note.* k=number of studies (i.e., papers); n=number of participants across studies; ES=number of effect sizes; ADHD=attention-deficit/hyperactivity disorder. Specific mental health outcomes were grouped into categories shown in the figure. Internalising problems includes internalising symptoms, internalising behavior, emotional symptoms, and trauma symptoms; conduct problems includes conduct disorder, conduct problems, antisocial, oppositional, or aggressive behavior, arrest or incarceration; externalising problems includes externalising symptoms or behavior; personality disorder includes borderline, paranoid, schizoid, schizotypal, histrionic, narcissistic, antisocial, avoidant, obsessive-compulsive and dependent personality disorders; psychopathology broad includes total psychopathology symptoms, the p-factor, any psychopathology disorder, and total behaviour problems (on the Strengths and Difficulties Questionnaire). Outcomes assessed in only one study (bulimia (37) and substance use disorder (54)) were not included in the moderator analysis.

The association between child maltreatment and mental health was generally similar across different mental health outcomes (Figure 3). Though stronger associations were found for autism symptoms, 4 of 5 of these effect sizes were from the ERA Study, and there was no statistically significant moderation after removing this cohort in a sensitivity analysis (Q_moderation=22.61; p-value=0.07).

#### Type of child maltreatment

**Figure 4:**
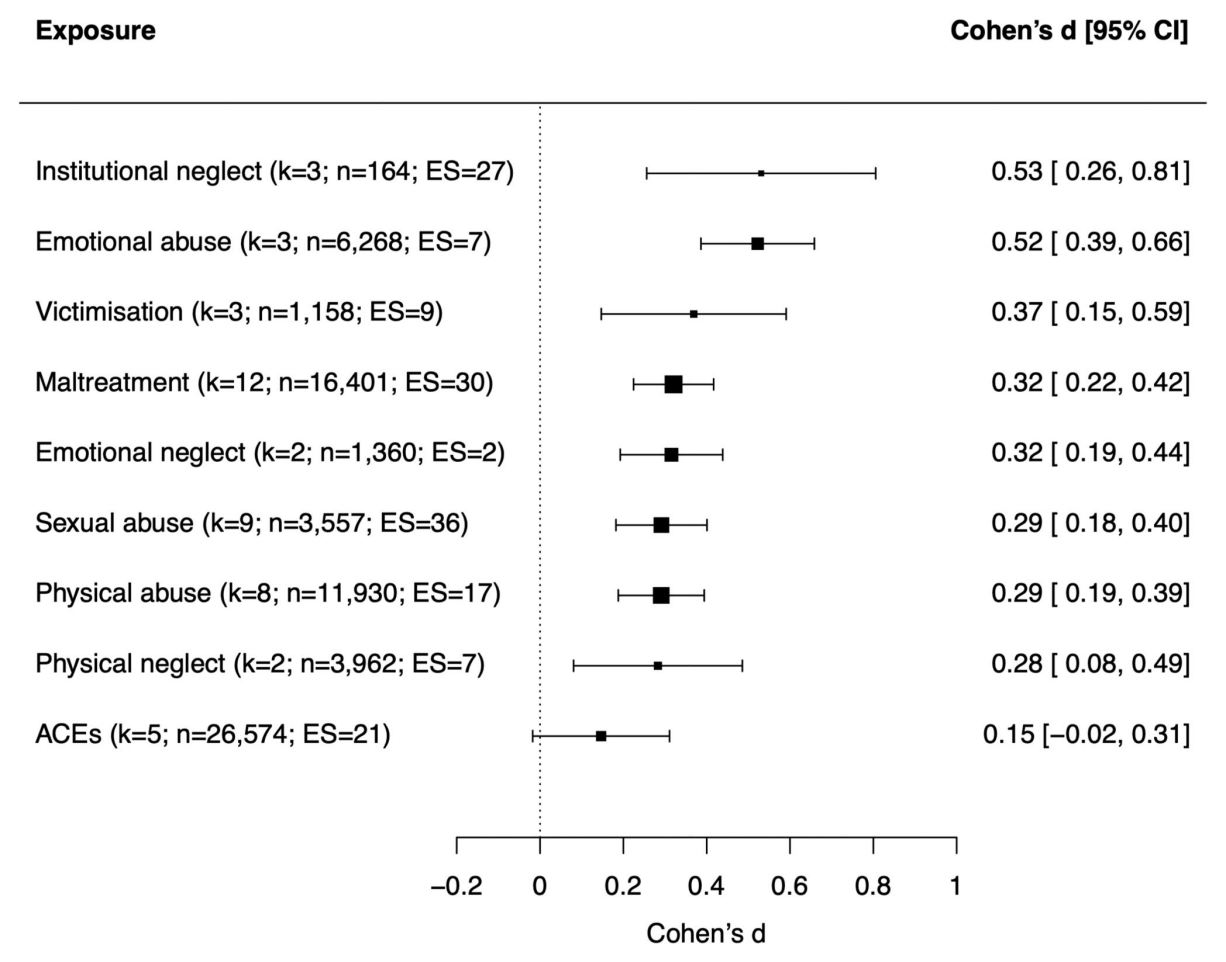
Meta-analytic associations between different types of child maltreatment and mental health problems Note. k=number of studies (i.e., papers); n=number of participants across studies; ES=number of effect sizes. Maltreatment includes assessment of multiple subtypes of abuse and/or neglect; victimisation includes assessment of maltreatment alongside other forms of victimisation (e.g., bullying); ACEs includes assessment of maltreatment alongside other forms of adverse childhood experiences.

The association between child maltreatment and mental health was moderated by the type of child maltreatment (Q_moderation=22.43; p-value=0.0042). As shown in Figure 4, emotional abuse and institutional neglect were more strongly associated with mental health problems than various subtypes of maltreatment and/or broader composite measures of maltreatment and ACEs. Specifically, pairwise comparisons showed that emotional abuse was more strongly associated with mental health than physical abuse, sexual abuse, emotional neglect, and broader measures of maltreatment and ACEs, while institutional neglect was more strongly associated with mental health than ACEs. However, such findings should be interpreted with caution as the estimates for emotional abuse were only based on 3 studies and 7 effect sizes, and institutional neglect was assessed only in the ERA Study.

#### Measurement characteristics

##### Prospective versus retrospective measures of maltreatment

The association between child maltreatment and mental health was similar in studies assessing maltreatment using prospective versus retrospective measures (Table 1), with no significant moderation effect (Q_moderation=0.25; p-value=0.61). This was also the case after we conducted a sensitivity analysis excluding the (prospective) ERA Study on institutional neglect (Q_moderation=1.01, p-value=0.31). However, we were not able to test the independent associations between prospective versus retrospective measures of maltreatment and psychopathology, as this was not tested by any quasi-experimental study.

##### Shared rater

The meta-analytic association was present when child maltreatment and mental health outcomes were assessed through a shared rater (self-reports) or through different sources (Table 1), with no moderation effect (Q_moderation=0.47, p-value=0.49). The findings remained similar after excluding the ERA Study, which included different sources (Q_moderation=1.78, p-value=0.18).

##### Longitudinal vs cross-sectional assessment

The meta-analytic association was consistent when childhood maltreatment and mental health were assessed cross-sectionally (i.e., at the same time point) or longitudinally (Table 1; Q_moderation=1.41, p-value=0.23). This was also the case after excluding the longitudinal ERA Study (Q_moderation=0.45, p-value=0.50) and when restricting to longitudinal quasi-experimental studies controlling for pre-existing psychopathology (Table 1).

#### Sample characteristics

The meta-analytic association between child maltreatment and mental health was not moderated by sex (Q_moderation=2.91; p-value=0.09) or age at mental health assessment (Q_moderation=0.02; p-value=0.87). We did not examine moderation by race or ethnicity as the majority of studies (25 of 34) did not report this information.

#### Study quality

Lastly, the meta-analytic association between child maltreatment and mental health was not moderated by an overall measure of study quality (Q_moderation=0.15; p-value=0.70; see Table S8 for coding).

## Discussion

To our knowledge, this is the first meta-analysis to examine the relationship between childhood maltreatment and mental health in quasi-experimental studies. Across 34 studies that included over 54,000 individuals, our meta-analysis provides novel insights into causality, confounding, and specificity of the relationship between child maltreatment and mental health.

Regarding causality, we found that child maltreatment had a small association with mental health problems (Cohen’s d=0.31) after stringent quasi-experimental control for confounding. Notably, this association triangulated across multiple types of quasi-experimental methods with different assumptions and potential sources of bias, strengthening causal inference (57, 58). Although a number of included studies used retrospective measures of maltreatment, findings were consistent among studies using prospective measures, suggesting that recall bias does not fully explain the relationship between maltreatment and mental health. Furthermore, while some studies were cross-sectional, we also observed consistent results in longitudinal studies controlling for pre-existing mental health problems, suggesting that the findings are not due to reverse causation. Taken together, this evidence is consistent with a small causal contribution of maltreatment to mental health. Although small, these effects of maltreatment could have far-reaching consequences, given that mental health problems predict poor outcomes in major life domains, such as occupational attainment (59), physical health (60), and mortality (61).

Regarding confounding, we found that the association between child maltreatment and mental health in quasi-experimental adjusted models was substantially (45%) smaller than in unadjusted models. Of note, the unadjusted association was moderate in magnitude (Cohen’s d=0.56) which is similar to effect sizes reported in prior meta-analyses of non-quasi-experimental studies (Table S10). This reduction in effect size after quasi-experimental adjustment suggests that a large part of the overall relationship between child maltreatment and mental health is confounded by pre-existing risk factors for psychopathology. Future research is needed to identify the specific factors that elevate risk of psychopathology in maltreated children, which might include environmental adversities (e.g., socioeconomic disadvantage; 9) and genetic liability (10, 11, 19).

Regarding specificity of effects, we observed three key findings. First, quasi-experimental evidence suggested small causal effects of maltreatment on a broad range of mental health outcomes, including internalizing disorders (e.g., depression, anxiety, suicidality, self-harm), externalizing disorders (e.g., conduct problems, ADHD, alcohol and drug abuse), and psychosis, rather than a few specific outcomes. This finding supports evidence from non-quasi experimental studies (62–66), and suggests that maltreatment may affect broad factors underlying multiple disorders (e.g., altered brain structure and function (67, 68), emotional dysregulation (69), or information-processing biases (70)) rather than disorder-specific risk factors (29).

Second, all subtypes of maltreatment were associated with mental health problems, including experiences involving threat (e.g., physical, sexual, and emotional abuse) and deprivation (e.g., neglect), consistent with non-quasi experimental evidence (71). However, emotional abuse and institutional neglect were more strongly associated with mental health problems than some other types of maltreatment. The stronger association between emotional abuse and mental health problems was based on evidence from only 3 studies, but supports findings from non-quasi-experimental studies (1, 62, 72, 73). If causal, it might occur because parental criticism becomes internalized and directly leads to negative self-views and distress (62, 72). However, non-causal mechanisms are also possible: for example, because emotional abuse is particularly likely to co-occur with other forms of maltreatment (62, 73), stronger associations might reflect cumulative effects of other maltreatment types. The finding might also partly reflect recall bias, as two of the three included studies used retrospective reports of emotional abuse (31, 41), which might be particularly influenced by current psychopathology given the more subjective nature of the experience (compared to other maltreatment types, e.g., sexual or physical abuse). Institutional neglect (experienced in Romanian orphanages) may also be strongly associated with mental health problems because (i) it reflects a particularly extreme, severe, and pervasive form of trauma, and/or (ii) it occurred the first few years of life (e.g., between birth to 3 years), disrupting development of brain architecture and neurobiological systems that can affect psychopathology (74).

Third, we did not find that effect sizes differed between studies assessing child maltreatment prospectively versus retrospectively. This may seem surprising, as prospective and retrospective measures identify largely different groups of individuals (75) and retrospective reports have been found to be more strongly associated with psychopathology than prospective measures (64, 76). Nevertheless, the overall pattern of our findings (i.e., visually larger effect sizes for retrospective vs prospective measures) broadly supports these previous results. Such differences may be less pronounced in this meta-analysis because of heterogeneity between samples, as quasi-experimental data were not available to compare effect sizes between prospective versus retrospective measures *within* the same samples, as in previous non-quasi-experimental research (64, 76).

Our findings should be interpreted in the context of limitations. First, each of the quasi-experimental approaches involves potential sources of bias (detailed in Table S3). For example, family-based designs do not account for non-familial confounding, panel data designs can be affected by time-varying confounding, propensity score approaches are liable to unmeasured confounding more generally, and natural experiments may not examine generalisable exposures (e.g., institutional neglect may be qualitatively different to maltreatment in the home). While we therefore cannot entirely rule out unmeasured confounding, convergent findings across multiple quasi-experimental methods provide stronger support for causal inference than findings from any individual method (i.e., triangulation; 57). Second, the type of quasi-experimental method can be related to the type of maltreatment measure used. For example, twin and sibling difference studies primarily used retrospective self-reports to assess maltreatment (e.g., 28, 31, 35, 53), likely because prospectively collected parent reports and official records detect minimal within-pair variation in maltreatment (26, 77). In contrast, the natural experiment design of the ERA Study meant that official records were used to prospectively assess institutional neglect (as adoption out of the Romanian institutions was handled by the authorities). It was not possible to disentangle the type of quasi-experimental method from these measurement characteristics in a multivariate moderation analysis, because limited data were available for comparisons (e.g., only 4 of 84 effect sizes from twin/sibling studies did not use self-reports to assess maltreatment and mental health). As such, we cannot rule out the possibility that some true moderation effects of measurement or design features were not detected due to suppression by other correlated variables. Third, we cannot draw firm conclusions about the specific effects of maltreatment types, because poly-victimization is common and studies rarely controlled for other co-occurring forms of maltreatment. Fourth, due to a lack of available data, we could not examine whether the findings were moderated by key factors, such as the timing of maltreatment, the time interval between maltreatment and psychopathology, and race or ethnicity. Future quasi-experimental research is needed to address these questions (e.g., utilising cohorts with detailed temporal measures of maltreatment, and racially diverse samples). Finally, although our meta-analysis focused on quasi-experimental studies, other designs can also strengthen causal inference about the effects of maltreatment, such as experimental animal studies (78) or high-quality prospective longitudinal studies (79–81).

Our findings have implications for research, clinical practice, and public health. Regarding future research, these findings highlight the importance of adopting rigorous quasi-experimental methods to test potentially causal effects of childhood maltreatment. Observational studies using conventional multiple regression approaches are likely to over-estimate the causal effects of maltreatment, and caution should thus be applied when interpreting such findings. Of course, quasi-experimental methods may not completely alleviate bias, and researchers should also take steps to further strengthen causal inference, such as using longitudinal designs and prospective measures incorporating multiple informants. Future research capitalising on these methods should provide valuable insights into the effects of child maltreatment on other outcomes (e.g., physical health, socioeconomic outcomes) and can be used to inform estimates of the economic costs of maltreatment (82, 83).

Regarding clinical practice, to minimise risk of psychopathology in individuals exposed to maltreatment, interventions should adopt a holistic approach that addresses both the maltreatment experience (e.g., via trauma-focused cognitive-behavioural therapy; 84) as well as wider risk factors for mental illness. Regarding public health, interventions that can prevent maltreatment (e.g., home visitation programs for high-risk families; 85, 86) should also prevent a proportion of cases of mental health problems in the population. As such, investing in these interventions is not only essential for children’s welfare, but could prevent long-term financial costs and suffering due to mental illness.

## Supplementary Material

Supplementary Material

## Figures and Tables

**Table 1:** Moderation of the quasi-experimental association between child maltreatment and mental health by assessment characteristics.

Moderator	k	ES	Cohen’s d (95% CI)
*Retrospective vs. prospective measures of maltreatment*			
Retrospective measures	23	109	0.32 (0.24-0.39)
Prospective measures	12	47	0.29 (0.19-0.39)
Prospective measures (excluding ERA)	9	20	0.25 (0.15-0.36)
*Shared rater vs. different sources*			
Shared rater (self-reports)	26	115	0.32 (0.25-0.39)
Different sources	9	41	0.28 (0.17-0.39)
Different sources (excluding ERA)	6	14	0.23 (0.10-0.35)
*Cross-sectional vs. longitudinal assessment*			
Cross-sectional	21	103	0.28 (0.19-0.36)
Longitudinal	13	53	0.36 (0.25-0.46)
Longitudinal (excluding ERA)	10	26	0.33 (0.21-0.44)
Longitudinal (controlling for earlier psychopathology)	8	37	0.34 (0.16-0.52)

Note. k=number of studies (i.e., papers); ES=number of effect sizes.

## References

[1] Nelson J, Klumparendt A, Doebler P, Ehring T (2017). Childhood maltreatment and characteristics of adult depression: meta-analysis. The British Journal of Psychiatry.

[2] Li M, D’arcy C, Meng X (2016). Maltreatment in childhood substantially increases the risk of adult depression and anxiety in prospective cohort studies: systematic review, meta-analysis, and proportional attributable fractions. Psychol Med.

[3] Varese F, Smeets F, Drukker M, Lieverse R, Lataster T, Viechtbauer W (2012). Childhood adversities increase the risk of psychosis: a meta-analysis of patient-control, prospective-and cross-sectional cohort studies. Schizophr Bull.

[4] Angelakis I, Gillespie EL, Panagioti M (2019). Childhood maltreatment and adult suicidality: a comprehensive systematic review with meta-analysis. Psychol Med.

[5] Liu RT, Scopelliti KM, Pittman SK, Zamora AS (2018). Childhood maltreatment and non-suicidal self-injury: a systematic review and meta-analysis. Lancet Psychiatry.

[6] Langevin R, Marshall C, Wallace A, Gagné M-E, Kingsland E, Temcheff C (2021). Disentangling the associations between attention deficit hyperactivity disorder and child sexual abuse: a systematic review. Trauma, Violence, Abuse.

[7] Maniglio R (2015). Significance, nature, and direction of the association between child sexual abuse and conduct disorder: A systematic review. Trauma, Violence, Abuse.

[8] Halpern SC, Schuch FB, Scherer JN, Sordi AO, Pachado M, Dalbosco C (2018). Child maltreatment and illicit substance abuse: A systematic review and meta-analysis of longitudinal studies. Child Abuse Review.

[9] Sidebotham P, Golding J, Team AS (2001). Child maltreatment in the “Children of the Nineties”: A longitudinal study of parental risk factors. Child Abuse Neglect.

[10] Sallis HM, Croft J, Havdahl A, Jones HJ, Dunn EC, Smith GD (2020). Genetic liability to schizophrenia is associated with exposure to traumatic events in childhood. Psychol Med.

[11] Ratanatharathorn A, Koenen KC, Chibnik LB, Weisskopf MG, Rich-Edwards JW, Roberts AL (2021). Polygenic risk for autism, attention-deficit hyperactivity disorder, schizophrenia, major depressive disorder, and neuroticism is associated with the experience of childhood abuse. Mol Psychiatry.

[12] Baldwin JR, Arseneault L, Odgers C, Belsky DW, Matthews T, Ambler A (2016). Childhood bullying victimization and overweight in young adulthood: A cohort study. Psychosom Med.

[13] Schoeler T, Duncan L, Cecil CM, Ploubidis GB, Pingault J-B (2018). Quasi-experimental evidence on short-and long-term consequences of bullying victimization: a meta-analysis. Psychological Bulletin.

[14] Jami ES, Hammerschlag AR, Bartels M, Middeldorp CM (2021). Parental characteristics and offspring mental health and related outcomes: a systematic review of genetically informative literature. Translational Psychiatry.

[15] Sonuga-Barke EJ, Kennedy M, Kumsta R, Knights N, Golm D, Rutter M (2017). Child-to-adult neurodevelopmental and mental health trajectories after early life deprivation: the young adult follow-up of the longitudinal English and Romanian Adoptees study. The Lancet.

[16] Martens EP, Pestman WR, de Boer A, Belitser SV, Klungel OH (2008). Systematic differences in treatment effect estimates between propensity score methods and logistic regression. Int J Epidemiol.

[17] Hughes K, Bellis MA, Hardcastle KA, Sethi D, Butchart A, Mikton C (2017). The effect of multiple adverse childhood experiences on health: a systematic review and meta-analysis. Lancet Public Health.

[18] Thornberry TP, Henry KL, Ireland TO, Smith CA (2010). The causal impact of childhood-limited maltreatment and adolescent maltreatment on early adult adjustment. J Adolesc Health.

[19] Warrier V, Kwong AS, Luo M, Dalvie S, Croft J, Sallis HM (2021). Gene–environment correlations and causal effects of childhood maltreatment on physical and mental health: a genetically informed approach. Lancet Psychiatry.

[20] Viechtbauer W (2010). Conducting meta-analyses in R with the metafor package. Journal of Statistical Software.

[21] Harrer M, Cuijpers P, Furukawa T, Ebert DD (2019). dmetar: Companion R Package For The Guide ‘Doing Meta-Analysis in R’. R package version 009000.

[22] Assink M, Wibbelink CJ (2016). Fitting three-level meta-analytic models in R: A step-by-step tutorial. The Quantitative Methods for Psychology.

[23] Rodgers MA, Pustejovsky JE (2021). Evaluating meta-analytic methods to detect selective reporting in the presence of dependent effect sizes. Psychol Methods.

[24] Simonsohn U, Nelson LD, Simmons JP (2014). P-curve: a key to the file-drawer. J Exp Psychol Gen.

[25] Stern A, Agnew-Blais J, Danese A, Fisher HL, Jaffee SR, Matthews T (2018). Associations between abuse/neglect and ADHD from childhood to young adulthood: a prospective nationally-representative twin study. Child Abuse Neglect.

[26] Dinkler L, Lundström S, Gajwani R, Lichtenstein P, Gillberg C, Minnis H (2017). Maltreatment-associated neurodevelopmental disorders: a co-twin control analysis. J Child Psychol Psychiatry.

[27] Baldwin JR, Arseneault A, Caspi A, Moffitt TE, Fisher HL, Odgers CL (2019). Adolescent victimization and self-injurious thoughts and behaviors: A genetically sensitive cohort study. J Am Acad Child Adolesc Psychiatry.

[28] Magnusson Å, Lundholm C, Göransson M, Copeland W, Heilig M, Pedersen N (2012). Familial influence and childhood trauma in female alcoholism. Psychol Med.

[29] Schaefer JD, Moffitt TE, Arseneault L, Danese A, Fisher HL, Houts R (2017). Adolescent victimization and early-adult psychopathology: Approaching causal inference using a longitudinal twin study to rule out non-causal explanations. Clin Psychol Sci.

[30] Alemany S, Goldberg X, van Winkel R, Gastó C, Peralta V, Fañanás L (2013). Childhood adversity and psychosis: examining whether the association is due to genetic confounding using a monozygotic twin differences approach. Eur Psychiatry.

[31] Bornovalova MA, Huibregtse BM, Hicks BM, Keyes M, McGue M, Iacono W (2013). Tests of a direct effect of childhood abuse on adult borderline personality disorder traits: a longitudinal discordant twin design. Journal of abnormal psychology.

[32] Capusan AJ, Kuja-Halkola R, Bendtsen P, Viding E, McCrory E, Marteinsdottir I (2016). Childhood maltreatment and attention deficit hyperactivity disorder symptoms in adults: a large twin study. Psychol Med.

[33] Young-Wolff KC, Kendler K, Ericson M, Prescott C (2011). Accounting for the association between childhood maltreatment and alcohol-use disorders in males: a twin study. Psychol Med.

[34] Berenz EC, Amstadter AB, Aggen SH, Knudsen GP, Reichborn-Kjennerud T, Gardner CO (2013). Childhood trauma and personality disorder criterion counts: a co-twin control analysis. Journal of abnormal psychology.

[35] Nelson EC, Heath AC, Lynskey MT, Bucholz KK, Madden PA, Statham DJ (2006). Childhood sexual abuse and risks for licit and illicit drug-related outcomes: a twin study. Psychol Med.

[36] Dinwiddie S, Heath AC, Dunne MP, Bucholz KK, Madden PA, Slutske WS (2000). Early sexual abuse and lifetime psychopathology: a co-twin-control study. Psychol Med.

[37] Kendler KS, Bulik CM, Silberg J, Hettema JM, Myers J, Prescott CA (2000). Childhood sexual abuse and adult psychiatric and substance use disorders in women: an epidemiological and cotwin control analysis. JAMA Psychiatry.

[38] Nelson EC, Heath AC, Madden PA, Cooper ML, Dinwiddie SH, Bucholz KK (2002). Association between self-reported childhood sexual abuse and adverse psychosocial outcomes: results from a twin study. Arch Gen Psychiatry.

[39] Schwartz JA, Wright EM, Valgardson BA (2019). Adverse childhood experiences and deleterious outcomes in adulthood: A consideration of the simultaneous role of genetic and environmental influences in two independent samples from the United States. Child Abuse Neglect.

[40] Barrigón ML, Diaz FJ, Gurpegui M, Ferrin M, Salcedo MD, Moreno-Granados J (2015). Childhood trauma as a risk factor for psychosis: A sib-pair study. J Psychiatr Res.

[41] Kullberg M-L, Van Schie C, Van Sprang E, Maciejewski D, Hartman CA, Van Hemert B (2021). It is a family affair: individual experiences and sibling exposure to emotional, physical and sexual abuse and the impact on adult depressive symptoms. Psychol Med.

[42] Lynch SK, Turkheimer E, D’Onofrio BM, Mendle J, Emery RE, Slutske WS (2006). A genetically informed study of the association between harsh punishment and offspring behavioral problems. J Fam Psychol.

[43] Riggins-Caspers KM, Cadoret RJ, Knutson JF, Langbehn D (2003). Biology-environment interaction and evocative biology-environment correlation: Contributions of harsh discipline and parental psychopathology to problem adolescent behaviors. Behav Genet.

[44] Voith LA, Gromoske AN, Holmes MR (2014). Effects of cumulative violence exposure on children’s trauma and depression symptoms: A social ecological examination using fixed effects regression. J Child Adolesc Trauma.

[45] Beckett C, Bredenkamp D, Castle J, Groothues C, O’connor TG, Rutter M (2002). Behavior patterns associated with institutional deprivation: A study of children adopted from Romania. J Dev Behav Pediatr.

[46] Golm D, Maughan B, Barker ED, Hill J, Kennedy M, Knights N (2020). Why does early childhood deprivation increase the risk for depression and anxiety in adulthood? A developmental cascade model. J Child Psychol Psychiatry.

[47] Gerin MI, Viding E, Pingault JB, Puetz VB, Knodt AR, Radtke SR (2019). Heightened amygdala reactivity and increased stress generation predict internalizing symptoms in adults following childhood maltreatment. J Child Psychol Psychiatry.

[48] Zvara B, Meltzer-Brody S, Mills-Koonce W, Cox M, Investigators FLPK (2017). Maternal childhood sexual trauma and early parenting: Prenatal and postnatal associations. Infant and child development.

[49] Alvanzo AA, Storr CL, Reboussin B, Green KM, Mojtabai R, La Flair LN (2020). Adverse childhood experiences (ACEs) and transitions in stages of alcohol involvement among US adults: Progression and regression. Child Abuse Neglect.

[50] Obikane E, Shinozaki T, Takagi D, Kawakami N (2018). Impact of childhood abuse on suicide-related behavior: analysis using marginal structural models. J Affect Disord.

[51] Kugler KC, Guastaferro K, Shenk CE, Beal SJ, Zadzora KM, Noll JG (2019). The effect of substantiated and unsubstantiated investigations of child maltreatment and subsequent adolescent health. Child Abuse Neglect.

[52] Ma J, Grogan-Kaylor A, Lee SJ (2018). Associations of neighborhood disorganization and maternal spanking with children’s aggression: A fixed-effects regression analysis. Child Abuse Neglect.

[53] Lecei A, Decoster J, De Hert M, Derom C, Jacobs N, Menne-Lothmann C (2019). Evidence that the association of childhood trauma with psychosis and related psychopathology is not explained by gene-environment correlation: A monozygotic twin differences approach. Schizophr Res.

[54] Capusan AJ, Gustafsson PA, Kuja-Halkola R, Igelström K, Mayo LM, Heilig M (2021). Re examining the link between childhood maltreatment and substance use disorder: a prospective, genetically informative study. Mol Psychiatry.

[55] Li X, Huebner ES, Tian L (2021). Vicious cycle of emotional maltreatment and bullying perpetration/victimization among early adolescents: Depressive symptoms as a mediator. Soc Sci Med.

[56] Isumi A, Ochi M, Kato T, Fujiwara T (2021). Child maltreatment and mental health in middle childhood: a longitudinal study in Japan. Am J Epidemiol.

[57] Munafò MR, Davey-Smith G (2018). Robust research needs many lines of evidence. Nature.

[58] Baldwin JR, Degli Esposti M (2021). Triangulating evidence on the role of perceived versus objective experiences of childhood adversity in psychopathology. JCPP Advances.

[59] Egan M, Daly M, Delaney L (2015). Childhood psychological distress and youth unemployment: Evidence from two British cohort studies. Soc Sci Med.

[60] Momen NC, Plana-Ripoll O, Agerbo E, Benros ME, Børglum AD, Christensen MK (2020). Association between mental disorders and subsequent medical conditions. New England Journal of Medicine.

[61] Ploubidis GB, Batty GD, Patalay P, Bann D, Goodman A (2021). Association of early-life mental health with biomarkers in midlife and premature mortality: evidence from the 1958 British Birth Cohort. JAMA psychiatry.

[62] Cecil CA, Viding E, Fearon P, Glaser D, McCrory EJ (2017). Disentangling the mental health impact of childhood abuse and neglect. Child Abuse Neglect.

[63] Vachon DD, Krueger RF, Rogosch FA, Cicchetti D (2015). Assessment of the harmful psychiatric and behavioral effects of different forms of child maltreatment. JAMA psychiatry.

[64] Newbury JB, Arseneault L, Moffitt TE, Caspi A, Danese A, Baldwin JR (2018). Measuring childhood maltreatment to predict early-adult psychopathology: comparison of prospective informant-reports and retrospective self-reports. J Psychiatr Res.

[65] Scott KM, Smith DR, Ellis PM (2010). Prospectively ascertained child maltreatment and its association with DSM-IV mental disorders in young adults. Arch Gen Psychiatry.

[66] Edwards VJ, Holden GW, Felitti VJ, Anda RF (2003). Relationship between multiple forms of childhood maltreatment and adult mental health in community respondents: results from the adverse childhood experiences study. Am J Psychiatry.

[67] Gehred MZ, Knodt AR, Ambler A, Bourassa KJ, Danese A, Elliott ML (2021). Long-term neural embedding of childhood adversity in a population-representative birth cohort followed for 5 decades. Biol Psychiatry.

[68] Jenness JL, Peverill M, Miller AB, Heleniak C, Robertson MM, Sambrook KA (2021). Alterations in neural circuits underlying emotion regulation following child maltreatment: A mechanism underlying trauma-related psychopathology. Psychol Med.

[69] Weissman DG, Bitran D, Miller AB, Schaefer JD, Sheridan MA, McLaughlin KA (2019). Difficulties with emotion regulation as a transdiagnostic mechanism linking child maltreatment with the emergence of psychopathology. Dev Psychopathol.

[70] McLaughlin KA, Lambert HK (2017). Child trauma exposure and psychopathology: mechanisms of risk and resilience. Current opinion in psychology.

[71] Miller AB, Machlin L, McLaughlin KA, Sheridan MA (2021). Deprivation and psychopathology in the Fragile Families Study: A 15-year longitudinal investigation. J Child Psychol Psychiatry.

[72] McGee RA, Wolfe DA, Wilson SK (1997). Multiple maltreatment experiences and adolescent behavior problems: Adolescents’ perspectives. Dev Psychopathol.

[73] Arata CM, Langhinrichsen-Rohling J, Bowers D, O’Brien N (2007). Differential correlates of multi-type maltreatment among urban youth. Child Abuse Neglect.

[74] Dunn EC, Nishimi K, Powers A, Bradley B (2017). Is developmental timing of trauma exposure associated with depressive and post-traumatic stress disorder symptoms in adulthood?. J Psychiatr Res.

[75] Baldwin JR, Reuben A, Newbury JB, Danese A (2019). Agreement between prospective and retrospective measures of childhood maltreatment: a systematic review and meta-analysis. JAMA Psychiatry.

[76] Reuben A, Moffitt TE, Caspi A, Belsky DW, Harrington H, Schroeder F (2016). Lest we forget: comparing retrospective and prospective assessments of adverse childhood experiences in the prediction of adult health. J Child Psychol Psychiatry.

[77] Jaffee SR, Caspi A, Moffitt TE, Taylor A (2004). Physical maltreatment victim to antisocial child: evidence of an environmentally mediated process. Journal of Abnormal Psychology.

[78] Sanchez MM, Ladd CO, Plotsky PM (2001). Early adverse experience as a developmental risk factor for later psychopathology: evidence from rodent and primate models. Dev Psychopathol.

[79] Arseneault L, Cannon M, Fisher HL, Polanczyk G, Moffitt TE, Caspi A (2011). Childhood trauma and children’s emerging psychotic symptoms: a genetically sensitive longitudinal cohort study. Am J Psychiatry.

[80] Horwitz AV, Widom CS, McLaughlin J, White HR (2001). The impact of childhood abuse and neglect on adult mental health: A prospective study. J Health Soc Behav.

[81] Croft J, Heron J, Teufel C, Cannon M, Wolke D, Thompson A (2019). Association of trauma type, age of exposure, and frequency in childhood and adolescence with psychotic experiences in early adulthood. JAMA psychiatry.

[82] Baldwin JR (2021). The economic costs linked to adverse childhood experiences in Europe. Lancet Public Health.

[83] Hughes K, Ford K, Bellis MA, Glendinning F, Harrison E, Passmore J (2021). Health and financial costs of adverse childhood experiences in 28 European countries: a systematic review and meta-analysis. Lancet Public Health.

[84] Early Intervention Foundation (2020). Trauma-Focused Cognitive Behavioural Therapy.

[85] Olds D, Henderson CR, Cole R, Eckenrode J, Kitzman H, Luckey D (1998). Long-term effects of nurse home visitation on children’s criminal and antisocial behavior: 15-year follow-up of a randomized controlled trial. JAMA.

[86] MacMillan HL, Wathen CN, Barlow J, Fergusson DM, Leventhal JM, Taussig HN (2009). Interventions to prevent child maltreatment and associated impairment. The Lancet.

[87] Del Re A, Hoyt WT (2014). MAd-package: Meta-Analysis with Mean Differences.

