# Supplementary material for: Childhood maltreatment and mental health problems: A systematic review and meta-analysis of quasi-experimental studies
Source: Am J Psychiatry. Author manuscript; Available in PMC 2023 Feb 20. (PMC7614155; doi:10.1176/appi.ajp.20220174)
Supplement: Supplementary Material [file EMS159513-supplement-Supplementary_Material.docx]

**Supplement**

**Childhood maltreatment and mental health problems:**

**A systematic review and meta-analysis of quasi-experimental studies**

[Methods S1. Search terms used to conduct the systematic search 2](#_Toc106222045)

[Methods S2. Data extraction details 3](#_Toc106222046)

[Methods S3. Adapted Newcastle-Ottowa Quality Assessment Scale 5](#_Toc106222047)

[Methods S4. Methods used to prepare data for effect size conversion 8](#_Toc106222048)

[Table S1. PRISMA reporting checklist 9](#_Toc106222049)

[Table S2. MOOSE (Meta-analyses Of Observational Studies in Epidemiology) Checklist 12](#_Toc106222050)

[Table S3. Overview of quasi-experimental methods to study the relationship between child maltreatment and mental health 14](#_Toc106222051)

[Table S4. Observational designs that were excluded from the systematic review and meta-analysis. 17](#_Toc106222052)

[Table S5. Formulae for conversion from raw effect sizes to Cohen’s d 18](#_Toc106222053)

[Table S6. Effect sizes that could not be converted to Cohen’s d and were therefore excluded 19](#_Toc106222054)

[Table S7. Quasi-experimental studies testing the association between childhood maltreatment and mental health 20](#_Toc106222055)

[Table S8. Quality and risk of bias assessment for included studies 26](#_Toc106222056)

[Table S9. Individual effect sizes included in the meta-analysis 27](#_Toc106222057)

[Table S10. Meta-analytic effect sizes for the relationship between maltreatment and mental health outcomes reported in previous meta-analyses of non-quasi-experimental studies. 32](#_Toc106222058)

[Figure S1. Study selection procedure 33](#_Toc106222059)

[Figure S2. Meta-analysis of the unadjusted association between childhood maltreatment and mental health problems 34](#_Toc106222060)

[Figure S3. Funnel plots 35](#_Toc106222061)

[Figure S4. P-curve analysis across study-averaged effect sizes 36](#_Toc106222062)

[Figure S5. Leave-one-out analysis in which each cohort was omitted in turn from the meta-analysis 37](#_Toc106222063)

[Figure S6. Leave-one-out analysis in which each cohort was omitted in turn from the meta-analysis 38](#_Toc106222064)

## Methods S1. Search terms used to conduct the systematic search

((maltreatment) OR (child* abuse) OR (child* neglect) OR (child* trauma) OR (child* advers*) OR (harsh punishment) OR (institutional deprivation) OR (early life deprivation) OR (early life stress) OR (victim*) OR (institutionali#ation))

AND ((mental health) OR (mental illness) OR (psychopathol*) OR (psychiatric) OR (internali*) OR (externali*) OR (depress*) OR (anxi*) OR (panic) OR (obsessive compulsive) OR (self inj*) OR (self harm*) OR (suicid*) OR (eating disorder*) OR (schiz*) OR (psychotic) OR (psychosis*) OR (bipolar) OR (ADHD) OR (attention deficit hyperactivity disorder) OR (attention) OR (hyperactiv*) OR (neurodev*) OR (conduct) OR (antisocial) OR (anti social) OR (crim*) OR (substance) OR (alcohol) OR (drug) OR (cannabis))

AND ((twin*) OR (sibling*) OR (children of twins) OR (natural experiment) OR (adopt*) OR (propensity score) OR (inverse probability weight*) OR (matching) OR (fixed effects) OR (quasiexperiment* OR quasi experiment*) OR (causal*) OR (genetically sens*) OR (genetically inform*) OR (instrumental variable*) OR (interrupted time series analysis) OR (Mendelian randomi#ation) OR (regression discontinuity) OR (experience sampl*) OR (ecological momentary assessment*) OR (difference in difference*)) .mp

Note: The search was conducted in Ovid (for Embase, PsycINFO and Medline).

## Methods S2. Data extraction details

*Variables extracted*

We extracted data containing the following information:

- cohort details (cohort name, country of origin, sex distribution, sample racial or ethnic distribution)
- type of maltreatment
- maltreatment measure information (type of measure, informant, observational period, age at assessment, prospective or retrospective measure)
- mental health measure information (type of measure, informant, observational period, age at assessment)
- quasi-experimental method used
- unadjusted analysis information (sample size and covariates, if any)
- unadjusted effect size and standard error
- quasi-experimental adjusted analysis information (sample size and covariates, if any)
- quasi-experimental adjusted effect size and standard error
- study quality items (see eMethods 2)

*Extraction of effect sizes based on categorical variables*

If the maltreatment variable was categorical (e.g., with categories reflecting “none”, “moderate” and “severe” exposure), we extracted effect sizes for the difference in mental health outcomes between the most severely maltreated group (e.g. “severe maltreatment”) with the non-exposed group. Similarly, if the mental health variable was categorical (e.g., “no problems”, “moderate problems” or “severe problems”), we extracted effect sizes reflecting the risk/odds of having the most severe outcome.

*Effect sizes from the ERA Study*

For the ERA Study, we extracted effect sizes for the difference in mental health outcomes between Romanian adoptees who experienced less than 6 months in an institution compared to those who experienced more than 6 months in an institution. Because the duration of exposure to institutional neglect was not likely to be associated with characteristics of the child or biological family (as adoptive parents had little choice over which child they selected)^1^, the two groups are likely to be similar in background characteristics, and differ only on the duration of exposure to institutional neglect. We did not extract effect sizes for the comparison between Romanian adoptees and UK adoptees because these groups may differ in background characteristics that might confound associations between institutional neglect and mental health.

## Methods S3. Adapted Newcastle-Ottowa Quality Assessment Scale

We adapted the Newcastle-Ottowa Quality Assessment Scale to include specific questions addressing the ability of a study to strengthen causal inference about the role of childhood maltreatment in mental health (see below for full scale). This involved adding questions assessing the extent to which the study controlled for environmental and genetic confounders (see questions 5 and 6), whether maltreatment and mental health outcomes were reported by different informants (i.e., addressing shared rater [or common-method] variance; see question 8), and whether maltreatment and mental health outcomes were assessed concurrently or longitudinally (see question 9). We derived an overall quality score for each study by summing the results across all items.

**Representativeness**

1) Representativeness of the exposed cohort

a) truly representative of the average cohort in the community (1)

b) somewhat representative of the average cohort in the community (0.5)

c) selected group of users eg nurses, volunteers (0)

d) no description of the derivation of the cohort (0)

2) Selection of the non-exposed cohort

a) drawn from the same community as the exposed cohort (1)

b) drawn from a different source (0)

c) no description of the derivation of the non exposed cohort (0)

**Exposure**

3) Ascertainment of exposure (maltreatment)

a) validated measure (official record or instrument tested for validity and reliability) (1)

b) non-validated measure or no description (0)

**Comparability/confounding**

4) Demonstration that outcome of interest was not present prior to exposure, or control for pre-existing outcome

a) yes (1)

b) no (0)

5) Study accounts for the majority of environmental confounders (e.g. SES, parenting, other adversities [e.g. bullying], either by design or statistically accounting for wide range of measured variables)

yes (e.g., co-twin control study controlling for victimization and other adversities outside of the family; or propensity score study controlling for SES, parenting, bullying, or other closely related adversities) (1)

some but not all (e.g., co-twin control study not controlling for bullying or other adversities, or propensity score study controlling for some but not all environments specified above) (0.5)

no (0)

6) Study fully accounts for genetic confounding

a) yes (e.g., MZ twin design) (1)

somewhat (e.g. DZ twin design, sibling design, or control for polygenic score or family history of outcome) (0.5)

no (0)

**Outcome**

7) Assessment of outcome

a) validated measure (official record or instrument tested for validity and reliability) (1)

b) non-validated measure or no description (0)

8) Outcome and maltreatment exposure reported by different informants

1. yes (1)
2. no (i.e., same person reported maltreatment and outcome) (0)

9) Outcomes assessed cross-sectionally (same time point as maltreatment assessed)

a) no (assessment was longitudinal – i.e., after exposure) (1)

b) yes – cross-sectional study / outcome assessed concurrently to maltreatment (0)

**Attrition**

10) Adequacy of follow up of cohorts

a) complete follow up - all subjects accounted for (1)

b) subjects lost to follow up unlikely to introduce bias - small number lost - > 70 % follow up, or method to account for attrition employed) (1)

c) follow up rate < 70% and no description of those lost

d) no statement (0)

## Methods S4. Methods used to prepare data for effect size conversion

Below we detail the methods used to prepare data for effect size conversion from studies that did not report conventional effect sizes or standard errors. Where effect sizes could not be derived, we contacted authors to request the necessary information.

*Effect sizes*. For studies reporting means and standard deviations (of psychiatric symptoms) in maltreated and non-maltreated groups, we directly calculated Cohen’s d. For studies reporting the raw prevalence of mental health problems among maltreated and non-maltreated groups,^2-4^ we calculated odds ratios. For a study reporting effect sizes between a measure of victimization standardised to mean=0, SD=1 and mental health outcomes standardised to mean=100, SD=15,^5^ we divided the coefficients by 15 to obtain standardized betas.

*Standard errors*. For studies that reported 95% confidence intervals (CIs) and not standard errors,^2,5-17^ we converted CIs to standard errors in Excel using the formula: SE = (upper CI-lower CI)/3.92 for differences measures or logSE = ln(upper CI)-ln(lower CI)/3.92 for odds ratios.^18^ For studies that did not report standard errors or confidence intervals for extracted effect sizes^19-22^, we derived the standard errors using standard formulae.^23^

## Table S1. PRISMA reporting checklist

| **Section and Topic** | **Item #** | **Checklist item** | **Location where item is reported** |
| --- | --- | --- | --- |
| **TITLE** | | |  |
| Title | 1 | Identify the report as a systematic review. | 1 |
| **ABSTRACT** | | |  |
| Abstract | 2 | See the PRISMA 2020 for Abstracts checklist. | 2 |
| **INTRODUCTION** | | |  |
| Rationale | 3 | Describe the rationale for the review in the context of existing knowledge. | 4 |
| Objectives | 4 | Provide an explicit statement of the objective(s) or question(s) the review addresses. | 5 |
| **METHODS** | | |  |
| Eligibility criteria | 5 | Specify the inclusion and exclusion criteria for the review and how studies were grouped for the syntheses. | 6 |
| Information sources | 6 | Specify all databases, registers, websites, organisations, reference lists and other sources searched or consulted to identify studies. Specify the date when each source was last searched or consulted. | 6 |
| Search strategy | 7 | Present the full search strategies for all databases, registers and websites, including any filters and limits used. | Methods S1 |
| Selection process | 8 | Specify the methods used to decide whether a study met the inclusion criteria of the review, including how many reviewers screened each record and each report retrieved, whether they worked independently, and if applicable, details of automation tools used in the process. | 6 |
| Data collection process | 9 | Specify the methods used to collect data from reports, including how many reviewers collected data from each report, whether they worked independently, any processes for obtaining or confirming data from study investigators, and if applicable, details of automation tools used in the process. | 6-7 |
| Data items | 10a | List and define all outcomes for which data were sought. Specify whether all results that were compatible with each outcome domain in each study were sought (e.g. for all measures, time points, analyses), and if not, the methods used to decide which results to collect. | 6-7 |
|  | 10b | List and define all other variables for which data were sought (e.g. participant and intervention characteristics, funding sources). Describe any assumptions made about any missing or unclear information. | Methods S2 |
| Study risk of bias assessment | 11 | Specify the methods used to assess risk of bias in the included studies, including details of the tool(s) used, how many reviewers assessed each study and whether they worked independently, and if applicable, details of automation tools used in the process. | Methods S3 |
| Effect measures | 12 | Specify for each outcome the effect measure(s) (e.g. risk ratio, mean difference) used in the synthesis or presentation of results. | 7 |
| Synthesis methods | 13a | Describe the processes used to decide which studies were eligible for each synthesis (e.g. tabulating the study intervention characteristics and comparing against the planned groups for each synthesis (item #5)). | NA |
|  | 13b | Describe any methods required to prepare the data for presentation or synthesis, such as handling of missing summary statistics, or data conversions. | Methods S4, Table S5 |
|  | 13c | Describe any methods used to tabulate or visually display results of individual studies and syntheses. | NA |
|  | 13d | Describe any methods used to synthesize results and provide a rationale for the choice(s). If meta-analysis was performed, describe the model(s), method(s) to identify the presence and extent of statistical heterogeneity, and software package(s) used. | 7 |
|  | 13e | Describe any methods used to explore possible causes of heterogeneity among study results (e.g. subgroup analysis, meta-regression). | 8 |
|  | 13f | Describe any sensitivity analyses conducted to assess robustness of the synthesized results. | 8 |
| Reporting bias assessment | 14 | Describe any methods used to assess risk of bias due to missing results in a synthesis (arising from reporting biases). | 8 |
| Certainty assessment | 15 | Describe any methods used to assess certainty (or confidence) in the body of evidence for an outcome. | NA |
| **RESULTS** | | |  |
| Study selection | 16a | Describe the results of the search and selection process, from the number of records identified in the search to the number of studies included in the review, ideally using a flow diagram. | Figure S1 |
|  | 16b | Cite studies that might appear to meet the inclusion criteria, but which were excluded, and explain why they were excluded. | 7 |
| Study characteristics | 17 | Cite each included study and present its characteristics. | Table S7 |
| Risk of bias in studies | 18 | Present assessments of risk of bias for each included study. | Table S8 |
| Results of individual studies | 19 | For all outcomes, present, for each study: (a) summary statistics for each group (where appropriate) and (b) an effect estimate and its precision (e.g. confidence/credible interval), ideally using structured tables or plots. | Table S9; Figures 1-4 |
| Results of syntheses | 20a | For each synthesis, briefly summarise the characteristics and risk of bias among contributing studies. | 9-12 |
|  | 20b | Present results of all statistical syntheses conducted. If meta-analysis was done, present for each the summary estimate and its precision (e.g. confidence/credible interval) and measures of statistical heterogeneity. If comparing groups, describe the direction of the effect. | 9-12 |
|  | 20c | Present results of all investigations of possible causes of heterogeneity among study results. | 11-13 |
|  | 20d | Present results of all sensitivity analyses conducted to assess the robustness of the synthesized results. | 10 |
| Reporting biases | 21 | Present assessments of risk of bias due to missing results (arising from reporting biases) for each synthesis assessed. | Figure S4 |
| Certainty of evidence | 22 | Present assessments of certainty (or confidence) in the body of evidence for each outcome assessed. | 9-12 |
| **DISCUSSION** | | |  |
| Discussion | 23a | Provide a general interpretation of the results in the context of other evidence. | 13-15 |
|  | 23b | Discuss any limitations of the evidence included in the review. | 16 |
|  | 23c | Discuss any limitations of the review processes used. | 16 |
|  | 23d | Discuss implications of the results for practice, policy, and future research. | 17-18 |
| **OTHER INFORMATION** | | |  |
| Registration and protocol | 24a | Provide registration information for the review, including register name and registration number, or state that the review was not registered. | 6 |
|  | 24b | Indicate where the review protocol can be accessed, or state that a protocol was not prepared. | 6 |
|  | 24c | Describe and explain any amendments to information provided at registration or in the protocol. | NA |
| Support | 25 | Describe sources of financial or non-financial support for the review, and the role of the funders or sponsors in the review. | 2 |
| Competing interests | 26 | Declare any competing interests of review authors. | 2 |
| Availability of data, code and other materials | 27 | Report which of the following are publicly available and where they can be found: template data collection forms; data extracted from included studies; data used for all analyses; analytic code; any other materials used in the review. | 7 |

## Table S2. MOOSE (Meta-analyses Of Observational Studies in Epidemiology) Checklist

| **Recommendation** | **Pg. no.** |
| --- | --- |
| **Reporting background should include** | |
| Problem definition | 4 |
| Hypothesis statement | 5 |
| Description of study outcome(s) | 4 |
| Type of exposure or intervention used | 4 |
| Type of study designs used | 4 |
| Study population | 6 |
| **Reporting of search strategy should include** | |
| Qualifications of searchers (e.g. librarians and investigators) | 6-7 |
| Search strategy, including time period included in the synthesis and keywords | Methods S1 |
| Effort to include all available studies, including contact with authors | Methods S4 |
| Databases and registries searched | 6 |
| Search software used, name and version, including special features | 6 |
| Use of hand searching (e.g. reference lists of obtained articles) | Figure S1 |
| List of citations located and those excluded including justification | Table S6; 7 |
| Method of addressing articles published in languages other than English | NA (only English language included) |
| Method of handling abstracts and unpublished studies | NA (only published studies included) |
| Description of any contact with authors | NA |
| **Reporting methods should include** | |
| Description of relevance or appropriateness of studies assembled for assessing the hypothesis to be tested | Table S3 |
| Rationale for the selection and coding of data | 6-7 |
| Documentation of how data were classified and coded (eg, multiple raters, blinding, and interrater reliability) | 6-7 |
| Assessment of confounding | Methods S3 |
| Assessment of study quality, including blinding of quality assessors; stratification or regression on possible predictors of study results | Methods S3 |
| Assessment of heterogeneity | 8 |
| Description of statistical methods (eg, complete description of fixed or random effects models, justification of whether the chosen models account for predictors of study results, dose-response models, or cumulative meta-analysis) in sufficient detail to be replicated | 7-8 |
| Provision of appropriate tables and graphics | Figs 1-4; Figs S1-S6 |
| **Reporting of results should include** | |
| Graphic summarizing individual study estimates and overall estimate | Figure 1 |
| Table giving descriptive information for each study included | Table S7 |
| Results of sensitivity testing (eg, subgroup analysis) | 9-10 |
| Indication of statistical uncertainty of findings | 9-12 |
| **Reporting of discussion should include** | |
| Quantitative assessment of bias (eg, publication bias) | 13 |
| Justification for exclusion (eg, exclusion of non–English-language citations) | NA |
| Assessment of quality of included studies | Table S8 |
| **Reporting of conclusions should include** | |
| Consideration of alternative explanations for observed results | 12-16 |
| Generalization of the conclusions (ie, appropriate for the data presented and within the domain of the literature review) | 12-16 |
| Guidelines for future research | 17 |
| Disclosure of funding source | 2 |

## Table S3. Overview of quasi-experimental methods to study the relationship between child maltreatment and mental health

| **Method** | **Description** | **Key limitations** |
| --- | --- | --- |
| ***Family-based designs*** | |  |
| Twin differences design | Examines whether twins who differ for exposure to childhood maltreatment differ in mental health outcomes. Because twins share their genotype (either 100% or 50% of their segregating genes, depending on whether they are monozygotic [MZ] or dizygotic [DZ], respectively) and by definition, their shared environment, any within-twin pair association between maltreatment and mental health must be independent of confounding by genetic influences (100% for MZ twins and 50% for DZ twins) and the shared family environment. | Does not control for individual-level (or non-shared) confounding by design.  Twin differences in maltreatment can be difficult to identify using prospective parent reports or child protection records, which tend to give concordant results for both children in a family (e.g., ^24^) and therefore self-reports are often used, which might involve recall bias. |
| Sibling differences design | Examines whether siblings who differ for exposure to childhood maltreatment differ in mental health outcomes. Similar to the twin differences design, because siblings share 50% their segregating genes, and their family environment, any within-sibling pair association between maltreatment and mental health must be independent of confounding by 50% of genetic influences and the shared family environment. | Only accounts for 50% of genetic influences and thus does not fully account for genetic confounding.  Does not control for individual-level (or non-shared) confounding by design.  Often relies on self-reports to maximise within-sibling pair variation in maltreatment measures. |
| Children of Twins design | Among MZ twin parents and their children, examines whether the association between maltreatment perpetration (by the twin parent) and child mental health is stronger in parent-child pairs versus aunt/uncle-child pairs. This design capitalises on the fact that the child is as genetically related to their parent’s twin as they are to their own parent, but they only experience maltreatment by their own parent (and not by their parent’s twin). Therefore, if the child’s mental health is more strongly associated with their own parent’s maltreatment perpetration than their aunt/uncle’s maltreatment perpetration, it indicates an effect of maltreatment above genetic confounding and the extended family environment. | Does not control for confounding from the nuclear family environment unless multiple children-of-twins are included.  Does not control for individual-level confounding by design. |
| Adoption design | Examines whether maltreatment perpetrated by adoptive parents is associated with the adopted child’s mental health. Because adoptive parents are not genetically related to their adopted children, the adoption design controls for confounding due to passive gene-environment correlations. | Does not control for environmental confounding by design, or genetic confounding arising from evocative gene-environment correlations. Assumes birth and adoptive parents have not been matched for characteristics that may influence child outcomes. |
| ***Panel data designs*** | |  |
| Fixed-effects design | Using data with repeated measures of maltreatment and mental health, examines whether within-individual changes in maltreatment exposure over time are associated with changes in mental health. Because each individual acts as their own control, all unmeasured, time-invariant confounders (e.g., stable genetic and environmental factors) are controlled for. | Does not control for time-variant confounders.  Does not rule out reverse causation (i.e., that changes in mental health affect maltreatment).  Requires there to be individual variation in maltreatment exposure over time. |
| Random intercept cross-lagged panel model | Similar to the fixed-effects design, cross-lagged paths examine whether within-individual changes in maltreatment exposure over time are associated with changes in mental health. The random intercept accounts for stable between-subject differences (e.g., time-invariant factors). | Does not control for time-variant confounders.  Does not rule out reverse causation (i.e., that changes in mental health affect maltreatment).  Requires there to be individual variation in maltreatment exposure over time. |
| ***Natural experiment*** | |  |
| Natural experiment design | Examines the mental health effects of maltreatment that is caused by wider social or political processes and is not related to family or individual risk factors. Because exposure to maltreatment is not affected by family or individual risk factors, any association with mental health must be independent of these confounding factors. An example of a natural experiment is the English and Romanian Adoptee (ERA) Study, which was set up after the fall of the Romanian Ceausescu regime. During the Ceausescu regime, contraception and abortions were banned and there was widespread poverty throughout Romania. This resulted in thousands of babies being abandoned in orphanages, where they experienced severe institutional neglect. After the Ceausescu regime fell, institutionalised children were adopted into Western families and a random subsample were followed-up by the ERA Study. The situation provided an opportunity to assess the effects of institutional neglect because: (i) it was very unlikely that institutionalisation was due to pre-existing child characteristics, as the majority of children were placed in institutions during the first few weeks of life and institutionalisation was widespread; (ii) it was possible to estimate the duration of exposure to institutional neglect, and (iii) the duration of institutional neglect was not influenced by child characteristics because adoptive parents had little choice over which child they adopted.^1^ | May be difficult to disentangle the effects of the exposure from other co-occurring risk factors, leading to a possibility of the risk factor being misidentified. However, in the ERA Study, the duration of exposure to institutional neglect was not associated with other risk factors such as birthweight (a marker of prenatal risk)^25^ or weight at adoption (a marker of malnutrition)^26^.  The exposure may differ to more typical experiences of maltreatment (e.g., institutional neglect in the Romanian orphanages was arguably more severe than neglect occurring in a family environment). |
| ***Propensity score methods*** | |  |
| Propensity score matching | Examines whether mental health outcomes differ between maltreated and non-maltreated individuals statistically matched for confounding factors. This analytic procedure involves (1) estimating a propensity score reflecting the risk of exposure to maltreatment based on measured background characteristics (i.e., confounders), (2) matching maltreated to non-maltreated individuals with a similar propensity score in order to reach an acceptable balance of confounders across maltreated and non-maltreated individuals, before (3) estimating the association between maltreatment and mental health within matched pairs. Any association between maltreatment and mental health should be independent of measured confounding factors included in the propensity score. | Only controls for unmeasured confounders to the extent they are associated with measured confounder. Measurement error in the confounder results in imperfect adjustment.  Can be sensitive to matching approach, with a trade-off between inexact matching (which can lead to residual confounding) and incomplete matching (which can limit generalisability and statistical power). |
| Inverse probability weighting | Examines whether maltreatment is associated with mental health problems after weighting the analysis to account for measured confounding factors. Similar to propensity score matching, inverse probability weighting involves (1) estimating a propensity score reflecting the risk of exposure to maltreatment based on measured background characteristic, and (2) estimating the association between maltreatment and mental health, after weighting for the inverse of the propensity score. Any association between maltreatment and mental health should be independent of measured confounding factors included in the propensity score. | Only controls for unmeasured confounders to the extent they are associated with measured confounder. Measurement error in the confounder results in imperfect adjustment.  Unstable for extreme weights. |

## Table S4. Observational designs that were excluded from the systematic review and meta-analysis.

| **Method** | **Reason for exclusion** |
| --- | --- |
| Bivariate twin model | Design does not aim to provide unbiased estimates of the effect of maltreatment on mental health, but rather decomposes the relationship into additive genetic, shared environmental, and unique environmental influences |
| Prospective longitudinal study | Potential for confounding by factors that are unmeasured (e.g., genetic influences), imperfectly measured, or which differ substantially between maltreated and non-maltreated individuals and therefore cannot effectively be controlled for. |
| Case-control study | As cases and controls are typically matched on only a few confounding variables, there is likely substantial potential for confounding by unmeasured factors, as well as imperfectly measured confounders used for matching. |

## Table S5. Formulae for conversion from raw effect sizes to Cohen’s d

| **Raw effect size type** | **Formula for conversion to Cohen’s d** | **Reference** |
| --- | --- | --- |
| Means and standard deviations | $d= \frac{\overline{X}_{1}- \overline{X}_{2}}{S_{within}}$  $S_{within}= \sqrt{\frac{{(n}_{1}-1)S_{1}^{2}+ {(n}_{2}-1)S_{2}^{2}}{n_{1}+n_{2}-2}}$ | ^27^ |
| Standardized beta / correlation coefficient | $d=\frac{2r}{\sqrt{1-r^{2}}}$ | ^28^ |
| Unstandardized beta | $d=\frac{B}{S_{pooled}}$  $S_{pooled}$= $\sqrt{\frac{s_{y}^{2} \left( N-1 \right)-B^{2}(\frac{n_{1}n_{2}}{n_{1}+ n_{2}})}{N-2}}$ | ^29^ |
| Log odds ratio | $d=LogOddsRatio \times\frac{\surd3}{\pi}$ | ^28^ |
| Relative risk | $LogOddsRatio=\frac{\log\left( 1-p \right)*RR}{1-RR*p}$  $d=LogOddsRatio \times\frac{\surd3}{\pi}$ | ^30^, ^28^ |
| Hazard ratio | $RR= \frac{1-e^{HRxln(1-p)}}{p}$ |  |

Note. $r$ = standardized beta or correlation coefficient; $B$ = unstandardized beta; $s_{y}$= standard deviation of the outcome variable; 𝑁 = total sample size; $n_{1}$ and $n_{2}$ = sample size in the exposed and unexposed groups (with equal group sizes estimated for a continuous independent variable); $RR$= relative risk; $p$=the prevalence rate in the unexposed individuals, calculated from results presented in the relevant paper^15,31^ and $HR$= hazard ratio.

## Table S6. Effect sizes that could not be converted to Cohen’s d and were therefore excluded

| **Reference** | **Study type** | **Effect size type** | **Exposure** | **Outcome** | **Effect size** | **SE** | **P-value reported** |
| --- | --- | --- | --- | --- | --- | --- | --- |
| Thornberry et al. (2010) | Propensity score matching | Negative binomial regression coefficient | Child-limited maltreatment | General offending | 0.04 | 0.25 | >0.05 |
| Thornberry et al. (2010) | Propensity score matching | Negative binomial regression coefficient | Child-limited maltreatment | Violent crime | -0.19 | 0.25 | >0.05 |
| Thornberry et al. (2010) | Propensity score matching | Negative binomial regression coefficient | Child-limited maltreatment | Problem alcohol use | 0.30 | 0.21 | >0.05 |
| Thornberry et al. (2010) | Propensity score matching | Negative binomial regression coefficient | Child-limited maltreatment | Problem drug use | 0.73 | 0.28 | <0.05 |
| Thornberry et al. (2010) | Propensity score matching | Negative binomial regression coefficient | Adolescent maltreatment | General offending | 0.72 | 0.30 | <0.05 |
| Thornberry et al. (2010) | Propensity score matching | Negative binomial regression coefficient | Adolescent maltreatment | Violent crime | 0.60 | 0.26 | <0.05 |
| Thornberry et al. (2010) | Propensity score matching | Negative binomial regression coefficient | Adolescent maltreatment | Problem alcohol use | 0.85 | 0.27 | <0.01 |
| Thornberry et al. (2010) | Propensity score matching | Negative binomial regression coefficient | Adolescent maltreatment | Problem drug use | 1.32 | 0.33 | <0.01 |
| Warrier et al. (2021) | Mendelian randomisation | Unstandardised beta coefficient | Childhood maltreatment | Depression | 0.598 | 0.145 | 3.63E-05 |
| Warrier et al. (2021) | Mendelian randomisation | Unstandardised beta coefficient | Childhood maltreatment | Schizophrenia | 1.167 | 0.268 | 1.35E-05 |
| Warrier et al. (2021) | Mendelian randomisation | Unstandardised beta coefficient | Childhood maltreatment | ADHD | 1.04 | 0.362 | 4.02E-03 |
| Warrier et al. (2021) | Mendelian randomisation | Unstandardised beta coefficient | Childhood maltreatment | Autism | 0.359 | 0.29 | 2.16E-01 |
| Warrier et al. (2021) | Mendelian randomisation | Unstandardised beta coefficient | Childhood maltreatment | Bipolar disorder | 0.563 | 0.298 | 5.90E-02 |

## Table S7. Quasi-experimental studies testing the association between childhood maltreatment and mental health

| **Reference** | **Cohort name/description, country** | **QE method** | **N (QE-adjusted; unadjusted** | **Maltreatment type** | **Age at maltreatment assessment** | **Maltreatment measure** | **Mental health outcome(s)** | **Age at mental health assessment** | **Mental health measure** |
| --- | --- | --- | --- | --- | --- | --- | --- | --- | --- |
| **MZ twin differences** | | | | | | | | | |
| Stern et al. (2018) | E-Risk Longitudinal Twin Study (E-Risk), UK | MZ twin differences | 1100; NA | Victimization | 5, 7, 10, 12 (prospective); 18 (retrospective) | Interview (parent); interview (self) | ADHD | 5, 7, 10, 12; 18 | Interview (parent, teacher); interview (self) |
| Dinkler et al. (2017) | Child and Adolescent Twin Study in Sweden (CATSS), Sweden | MZ twin differences | 3568; 8166 | Maltreatment | 9 (prospective) | Interview (parent) | ADHD; autism | 9 | Interview (parent) |
| Baldwin et al. (2019) | E-Risk Longitudinal Twin Study (E-Risk), UK | MZ twin differences | 1100; 2055 | Victimization | 18 (retrospective) | Interview (self) | Suicidal ideation; self harm; suicide attempt | 18 | Interview (self) |
| Magnusson et al. (2012) | The Study of Twin Adults: Genes and Environment (STAGE), Sweden | MZ twin differences | 44; 13595 | Emotional neglect; physical abuse; sexual abuse | 33.5 (retrospective) | Questionnaire (self) | Alcohol dependence | 33.5 | Questionnaire (self) |
| Schaefer et al. (2017) | E-Risk Longitudinal Twin Study (E-Risk), UK | MZ twin differences | 1158; 2062 | Victimization | 18 (retrospective) | Interview (self) | P-factor; internalising; externalising; thought disorder | 18 | Interview (self) |
| Alemany et al. (2013) | Cross-sectional study of adult twins from Catalonia, Spain | MZ twin differences | 170; 226 | ACEs | 33.8 (retrospective) | Questionnaire (self) | Positive psychotic experiences; negative psychotic experiences | 33.8 | Questionnaire (self) |
| Bornovalova et al. (2013) | Minnesota Twin Family Study (MTFS), USA | MZ twin differences | 1792; 2764 | Abuse; emotional abuse; physical abuse; sexual abuse | 20, 24, 29 (retrospective) | Interview (self) | Borderline personality disorder | 24.9 | Questionnaire (self) |
| Capusan et al. (2016) | The Study of Twin Adults: Genes and Environment (STAGE), Sweden | MZ twin differences | 940; 17711 | Maltreatment; emotional neglect; physical neglect; physical abuse; sexual abuse; abuse; neglect | 33.8 (retrospective) | Questionnaire (self) | ADHD | 33.8 | Questionnaire (self) |
| Lecei et al. (2019) | twinssCan Study, Belgium | MZ twin differences | 266; 266 | Maltreatment | 18.2 (retrospective) | Questionnaire (self) | Total psychopathology; psychosis; anxiety; depression | 18.2 | Questionnaire (self) |
| Young-Wolff et al. (2011) | Virginia Adult Twin Study of Psychiatric and Substance Use Disorders (VATSPSUD), USA | MZ twin differences | 174; 3527 | Maltreatment | 35 (retrospective) | Interview (self) | Alcohol abuse/ dependence | 35 | Interview (self) |
| **Twin differences** | | | | | | | | | |
| Berenz et al. (2013) | Norwegian Twin Registry (NTR), Norway | Twin differences | 616; 2780 | ACEs | 28.2 (retrospective) | Interview (self) | Personality disorders: paranoid; schizoid; schizotypal; histrionic; narcissistic; borderline; antisocial; avoidant; obsessive compulsive; dependent | 28.2 | Interview (self) |
| Nelson et al. (2006) | Australian Twin Register young adult cohort, Australia | Twin differences | 280; NA | Sexual abuse | 29.9 (retrospective) | Interview (self) | Dependence/ abuse: cannabis, opioids; sedatives; stimulants; cocaine; any illicit drug; non-cannabis illicit drug | 29.9 | Interview (self) |
| Dinwiddie et al. (2000) | Australian National Health and Medical Research Council (NH&MRC) Twin Register, Australia | Twin differences | 75; 3180 | Sexual abuse | 44.1 (retrospective) | Interview (self) | Depression; suicidal ideation; suicide attempt; panic disorder; social phobia; alcohol dependence; conduct disorder; psychopathology any | 44.1 | Interview (self) |
| Kendler et al. (2000) | Virginia Twin Registry (VTR), USA | Twin differences | 133; 1403 | Sexual abuse | 32.7 (retrospective) | Questionnaire (self) | Depression; GAD; alcohol dependence; drug dependence; bulimia | 37.6 | Interview (self) |
| Nelson et al. (2002) | Australian Twin Register young adult cohort, Australia | Twin differences | 73; NA | Sexual abuse | 29.9 (retrospective) | Interview (self) | Depression; suicide attempt; conduct disorder; alcohol dependence; social anxiety | 29.9 | Interview (self) |
| Schwartz et al. (2019) | Midlife in the United States (MIDUS), USA | Twin differences | 862; 862 | ACEs | 46, 50 (retrospective) | Interview (self) | Depression; antisocial behaviour | 50 | Interview (self) |
| **Sibling differences** | | | | | | | | | |
| Barrigon et al. (2015) | Cross-sectional study of patients with psychosis and unaffected siblings from Granada and Jaen, Spain | Sibling differences | 98; NA | Maltreatment | 31.7 (retrospective) | Interview (self) | Psychosis | 31.7 | Interview (self) |
| Kullberg et al. (2020) | Netherlands Study of Depression and Anxiety (NESDA), Netherlands | Sibling differences | 636; 636 | Emotional abuse; physical abuse; sexual abuse | 49.7 (retrospective) | Questionnaire (self) | Depression; anxiety | 49.7 | Questionnaire (self) |
| Schwartz et al. (2019) | Add Health, USA | Sibling differences | 3112; 3112 | ACEs | 16.1, 23, 30 (retrospective) | Interview (self) | Depression | 30 | Interview (self) |
| Capusan et al. (2021) | Official record study of participants from Östergötland, Sweden | Sibling differences | 865; 3887 | Maltreatment | 0-18 (prospective) | Medical record | Substance use disorder | 29.5 | Medical record |
| **Children of twins** | | | | | | | | | |
| Lynch et al. (2006) | Australian Twin Register children of twins, Australia | Children of twins | 2502; 1926 | Physical abuse | 25.1 (retrospective) | Interview (self) | Externalising behaviour; drug and alcohol use; internalising behaviour | 25.1 | Interview (self) |
| **Adoption design** | | | | | | | | | |
| Riggins-Caspers et al. (2003) | Cross-sectional study of adult adoptees from Iowa, USA | Adoption design | 150; NA | Physical abuse | 31.5 (retrospective) | Questionnaire (self) | Conduct disorder; oppositional behaviour | 31.5 | Interview (adoptive parent) |
| **Within-individual fixed-effects** | | | | | | | | | |
| Ma et al. (2018) | Fragile Families and Child Wellbeing Study (FFCWS), USA | Fixed effects | 2472; NA | Physical abuse | 3, 5 (prospective) | Questionnaire (parent) | Aggressive behaviour | 3,5 | Questionnaire (parent) |
| Voith et al. (2014) | National Survey of Child and Adolescent Well-Being (NSCAW-I), USA | Fixed effects | 1022; NA | ACEs | 10.3, 12.2 (prospective) | Interview (mixed) | Trauma; depression | 10.3, 12.2 | Interview (self) |
| Isumi et al. (2021) | Adachi Child Health Impact of Living Difficulty (A-CHILD), Japan | Fixed effects | 2920; NA | Maltreatment | 6.5, 7.5, 9.5 (prospective) | Questionnaire (parent) | Behavioural difficulties | 6.5, 7.5, 9.5 | Questionnaire (parent) |
| **Random intercept cross-lagged panel model** | | | | | | | | | |
| Li et al. (2021) | Longitudinal study of students from schools in Guangdong, China, China | NA | 3742; 3742 | Emotional abuse | 9.9; 10.4; 10.9; 11.4 (prospective) | Questionnaire (self) | Depression | 10.4; 10.9; 11.4; 11.9 | Questionnaire (self) |
| **Natural experiment** | | | | | | | | | |
| Beckett et al. (2002) | English and Romanian Adoptees Study (ERA), UK/Romania | Natural experiment | 90; NA | Institutional neglect | 0-3.6 (prospective) | Government record | Self-injury | 6 | Interview (parent) |
| Golm et al. (2020) | English and Romanian Adoptees Study (ERA), UK/Romania | Natural experiment | 98; NA | Institutional neglect | 0-3.6 (prospective) | Government record | Depression; generalised anxiety | 23.9 | Questionnaire (parent) |
| Sonuga-Barke et al. (2017) | English and Romanian Adoptees Study (ERA), UK/Romania | Natural experiment | 148; NA | Institutional neglect | 0-3.6 (prospective) | Government record | ASD; inattention overactivity; emotional; conduct problem | 6; 11; 15; 24.1 | Questionnaire (parent) |
| **Propensity score matching** | | | | | | | | | |
| Thornberry et al. (2010) | Rochester Youth Development Study (RYDS), USA | Propensity score matching | 907; NA | Maltreatment | 14, 15, 16, 17, 18 (prospective) | CPS record | Arrest or incarceration; suicidal thoughts; depression | 22.7 | Interview (self) or crime record (for arrest) |
| Gerin et al. (2019) | Duke Neurogenetics Study (DNS), USA | Propensity score matching | 196; NA | Maltreatment | 19.5 (retrospective) | Questionnaire (self) | Internalising | 20.5 | Questionnaire (self) |
| Zvara et al. (2017) | Family Life Project (FLP), USA | Propensity score matching | 204; NA | Sexual abuse | 29.8 (retrospective) | Questionnaire (self) | Postnatal depression | 25.8 | Questionnaire (self) |
| **Inverse probability weighting** | | | | | | | | | |
| Kugler et al. (2019) | Female Adolescent Development Study, USA | Inverse probability weighting | 367; NA | Maltreatment | 14, 15, 16, 17, 18 (prospective) | CPS record | Drug use; depression | 19 | Questionnaire (self) |
| Alvanzo et al. (2020) | National Epidemiologic Survey on Alcohol and Related Conditions (NESARC), USA | Inverse probability weighting | 10396; 10396 | ACEs | 45.9; 46.5 (retrospective) | Interview (self) | Severe alcohol problems; moderate alcohol problems | 43.9; 44.5 | Interview (self) |
| Obikane et al. (2018) | The Japanese Study on Stratification, Health, Income, and Neighborhood (J-SHINE), Japan | Inverse probability weighting | 1896; 1896 | Physical abuse; physical neglect; maltreatment | 36.5 (retrospective) | Questionnaire (self) | Suicidal ideation; suicidal plan; suicide attempt | 36.5 | Questionnaire (self) |

Abbreviations: QE = quasi-experimental; MZ monozygotic; CPS = child protection services; ADHD = attention deficit/hyperactivity disorder; GAD = generalized anxiety disorder.

^1^ “Maltreatment” includes assessment of multiple subtypes of abuse and/or neglect (i.e., physical abuse, sexual abuse, emotional abuse, physical neglect, or emotional neglect); “victimization” includes assessment of any of forms of maltreatment alongside other types of victimization (e.g., bullying); “ACEs” includes assessment of any of forms of maltreatment alongside other adverse childhood experiences (e.g., domestic violence).

## Table S8. Quality and risk of bias assessment for included studies

| **Reference** | **Represent. (exposed)** | **Exposed & unexposed from same cohort** | **Validated MT assess.** | **Control for pre-existing MH** | **Control for environ. confound.** | **Control for genetic confound.** | **Validated MH assess** | **Different informants for MT & MH** | **Longitud. assess.** | **Retention >70%** | **Total quality score** |
| --- | --- | --- | --- | --- | --- | --- | --- | --- | --- | --- | --- |
| Alemany et al. (2013) | 0.5 | 1 | 1 | 0 | 0.5 | 1 | 1 | 0 | 0 | 1 | 6 |
| Alvanzo et al. (2020) | 1 | 1 | 0 | 0 | 0 | 0.5 | 1 | 0 | 0 | 1 | 4.5 |
| Baldwin et al. (2019) | 1 | 1 | 1 | 1 | 1 | 1 | 0 | 0 | 0 | 1 | 7 |
| Barrigon et al. (2015) | 0 | 1 | 0 | 0 | 0.5 | 0.5 | 1 | 0 | 0 | 0 | 3 |
| Beckett et al. (2002) | 1 | 1 | 1 | 1 | 1 | 1 | 0 | 1 | 1 | 1 | 9 |
| Berenz et al. (2013) | 1 | 1 | 1 | 0 | 0.5 | 0.5 | 1 | 0 | 0 | 0 | 5 |
| Bornovalova et al. (2013) | 1 | 1 | 1 | 0 | 0.5 | 1 | 1 | 0 | 0 | 1 | 6.5 |
| Capusan et al. (2016) | 1 | 1 | 1 | 0 | 0.5 | 1 | 1 | 0 | 0 | 1 | 6.5 |
| Capusan et al. (2021) | 0.5 | 1 | 1 | 0 | 0.5 | 0.5 | 1 | 1 | 1 | 1 | 7.5 |
| Dinkler et al. (2017) | 1 | 1 | 1 | 0 | 0.5 | 1 | 1 | 0 | 0 | 1 | 6.5 |
| Dinwiddie et al. (2000) | 0.5 | 1 | 0 | 0 | 0.5 | 0.5 | 1 | 0 | 0 | 1 | 4.5 |
| Gerin et al. (2019) | 0 | 1 | 1 | 0 | 0.5 | 0 | 1 | 0 | 1 | 0 | 4.5 |
| Golm et al. (2020) | 1 | 1 | 1 | 1 | 1 | 1 | 1 | 1 | 1 | 0 | 9 |
| Isumi et al. (2021) | 1 | 1 | 1 | 1 | 0.5 | 1 | 1 | 0 | 1 | 1 | 8.5 |
| Kendler et al. (2000) | 0.5 | 1 | 0 | 0 | 0.5 | 0.5 | 1 | 0 | 1 | 0 | 4.5 |
| Kugler et al. (2019) | 0 | 1 | 1 | 1 | 0.5 | 0 | 1 | 1 | 1 | 1 | 7.5 |
| Kullberg et al. (2020) | 0 | 1 | 1 | 0 | 0.5 | 0.5 | 1 | 0 | 0 | 0 | 4 |
| Lecei et al. (2019) | 0.5 | 1 | 1 | 0 | 0.5 | 1 | 1 | 0 | 0 | 1 | 6 |
| Li et al. (2021) | 1 | 1 | 1 | 1 | 0.5 | 1 | 1 | 0 | 1 | 1 | 8.5 |
| Lynch et al. (2006) | 0.5 | 1 | 0 | 0 | 0.5 | 0.5 | 1 | 0 | 0 | 1 | 4.5 |
| Ma et al. (2018) | 0.5 | 1 | 1 | 1 | 1 | 1 | 1 | 0 | 1 | 1 | 8.5 |
| Magnusson et al. (2012) | 0.5 | 1 | 1 | 0 | 0.5 | 1 | 1 | 0 | 0 | 0 | 5 |
| Nelson et al. (2002) | 1 | 1 | 0 | 0 | 0.5 | 1 | 1 | 0 | 0 | 0 | 4.5 |
| Nelson et al. (2006) | 1 | 1 | 0 | 0 | 0.5 | 0.5 | 1 | 0 | 0 | 0 | 4 |
| Obikane et al. (2018) | 1 | 1 | 0 | 1 | 0.5 | 0 | 1 | 0 | 0 | 0 | 4.5 |
| Riggins-Caspers et al. (2003) | 0 | 1 | 1 | 0 | 0 | 0.5 | 0 | 1 | 0 | 1 | 4.5 |
| Schaefer et al. (2017) | 1 | 1 | 1 | 1 | 0.5 | 1 | 1 | 0 | 0 | 1 | 7.5 |
| Schwartz et al. (2019) | 1 | 1 | 0 | 0 | 0.5 | 0.5 | 1 | 0 | 1 | 1 | 6 |
| Sonuga-Barke et al. (2017) | 1 | 1 | 1 | 1 | 1 | 1 | 1 | 1 | 1 | 1 | 10 |
| Stern et al. (2018) | 1 | 1 | 1 | 0 | 0.5 | 1 | 1 | 1 | 0 | 1 | 7.5 |
| Thornberry et al. (2010) | 0.5 | 1 | 1 | 0 | 0.5 | 0.5 | 1 | 1 | 1 | 1 | 7.5 |
| Voith et al. (2014) | 0 | 1 | 1 | 1 | 0.5 | 1 | 1 | 1 | 1 | 0 | 7.5 |
| Young-Wolff et al. (2011) | 0.5 | 1 | 0 | 0 | 0.5 | 1 | 1 | 0 | 0 | 0 | 4 |
| Zvara et al. (2017) | 1 | 1 | 1 | 0 | 1 | 0 | 1 | 0 | 0 | 0 | 5 |

Note. MT=maltreatment; MH=mental health; assess.=assessment; confound=confounders.This table shows a single quality score for each study, but occasionally the scores varied within a single study (e.g., if some outcomes were assessed longitudinally and others cross-sectionally). Here we have reported the maximum score for each study.

## Table S9. Individual effect sizes included in the meta-analysis

| **Reference** | **Cohort name/description** | **Maltreatment type** | **Mental health outcome** | **Cohen’s d** | **95% CI** |
| --- | --- | --- | --- | --- | --- |
| Dinwiddie et al. (2000) | ATR (females) | Sexual abuse | Depression | 0.197 | -0.180-0.575 |
| Dinwiddie et al. (2000) | ATR (females) | Sexual abuse | Suicidal ideation | 0.252 | -0.065-0.569 |
| Dinwiddie et al. (2000) | ATR (females) | Sexual abuse | Suicide attempt | 0.466 | -0.281-1.213 |
| Dinwiddie et al. (2000) | ATR (females) | Sexual abuse | Panic disorder | 0.382 | -0.211-0.975 |
| Dinwiddie et al. (2000) | ATR (females) | Sexual abuse | Social phobia | 0.224 | -0.476-0.923 |
| Dinwiddie et al. (2000) | ATR (females) | Sexual abuse | Alcohol dependence | 0.505 | -0.017-1.027 |
| Dinwiddie et al. (2000) | ATR (females) | Sexual abuse | Conduct disorder | 0.123 | -0.599-0.845 |
| Dinwiddie et al. (2000) | ATR (females) | Sexual abuse | Psychopathology any | 0.242 | -0.069-0.553 |
| Dinwiddie et al. (2000) | ATR (males) | Sexual abuse | Depression | 0.224 | -0.476-0.923 |
| Dinwiddie et al. (2000) | ATR (males) | Sexual abuse | Suicidal ideation | 0.940 | 0.110-1.770 |
| Dinwiddie et al. (2000) | ATR (males) | Sexual abuse | Alcohol dependence | 0.000 | -0.885-0.885 |
| Dinwiddie et al. (2000) | ATR (males) | Sexual abuse | Conduct disorder | 0.382 | -0.382-1.146 |
| Dinwiddie et al. (2000) | ATR (males) | Sexual abuse | Psychopathology any | 0.382 | -0.281-1.045 |
| Kendler et al. (2000) | VTR | Sexual abuse | Depression | 0.186 | -0.095-0.466 |
| Kendler et al. (2000) | VTR | Sexual abuse | GAD | 0.212 | -0.144-0.569 |
| Kendler et al. (2000) | VTR | Sexual abuse | Alcohol dependence | 0.574 | 0.066-1.081 |
| Kendler et al. (2000) | VTR | Sexual abuse | Drug dependence | 0.382 | -0.212-0.976 |
| Kendler et al. (2000) | VTR | Sexual abuse | Bulimia | 0.157 | -0.664-0.978 |
| Beckett et al. (2002) | ERA | Institutional neglect | Self injury | 1.219 | 0.084-2.353 |
| Nelson et al. (2002) | ATR (young adults) | Sexual abuse | Depression | 0.245 | 0.033-0.458 |
| Nelson et al. (2002) | ATR (young adults) | Sexual abuse | Suicide attempt | 0.554 | 0.174-0.934 |
| Nelson et al. (2002) | ATR (young adults) | Sexual abuse | Conduct disorder | 0.606 | 0.165-1.046 |
| Nelson et al. (2002) | ATR (young adults) | Sexual abuse | Alcohol dependence | 0.245 | 0.007-0.484 |
| Nelson et al. (2002) | ATR (young adults) | Sexual abuse | Social anxiety | 0.466 | 0.132-0.801 |
| Riggins-Caspers et al. (2003) | Iowa adoption study | Physical abuse | Conduct disorder | 0.387 | 0.059-0.715 |
| Riggins-Caspers et al. (2003) | Iowa adoption study | Physical abuse | Oppositional behaviour | 0.516 | 0.184-0.849 |
| Lynch et al. (2006) | ATR (CoT) | Physical abuse | Externalising behaviour | 0.303 | 0.179-0.426 |
| Lynch et al. (2006) | ATR (CoT) | Physical abuse | Drug and alcohol use | 0.343 | 0.220-0.467 |
| Lynch et al. (2006) | ATR (CoT) | Physical abuse | Internalising behaviour | 0.165 | 0.042-0.289 |
| Nelson et al. (2006) | ATR (young adults) | Sexual abuse | Cannabis dep. | 0.136 | -0.152-0.424 |
| Nelson et al. (2006) | ATR (young adults) | Sexual abuse | Opioids dep. | 1.032 | 0.212-1.852 |
| Nelson et al. (2006) | ATR (young adults) | Sexual abuse | Sedatives dep. | 0.829 | -0.016-1.674 |
| Nelson et al. (2006) | ATR (young adults) | Sexual abuse | Stimulants dep. | 0.302 | -0.108-0.712 |
| Nelson et al. (2006) | ATR (young adults) | Sexual abuse | Cocaine dep. | 0.382 | -0.382-1.146 |
| Nelson et al. (2006) | ATR (young adults) | Sexual abuse | Any illicit drug dep. | 0.318 | 0.036-0.599 |
| Nelson et al. (2006) | ATR (young adults) | Sexual abuse | Non-cannabis illicitdrug abuse | 0.430 | 0.037-0.823 |
| Thornberry et al. (2010) | RYDS | Maltreatment (0-11y) | Arrest or incarceration | 0.243 | -0.006-0.491 |
| Thornberry et al. (2010) | RYDS | Maltreatment (0-11y) | Suicidal thoughts | 0.369 | 0.067-0.672 |
| Thornberry et al. (2010) | RYDS | Maltreatment (0-11y) | Depression | 0.209 | 0.004-0.413 |
| Thornberry et al. (2010) | RYDS | Maltreatment (12-17y) | Arrest or incarceration | 0.408 | 0.105-0.711 |
| Thornberry et al. (2010) | RYDS | Maltreatment (12-17y) | Suicidal thoughts | 0.496 | 0.150-0.842 |
| Thornberry et al. (2010) | RYDS | Maltreatment (12-17y) | Depression | 0.197 | -0.044-0.438 |
| Young-Wolff et al. (2011) | VATSPSUD | Maltreatment | Alcohol abuse/dependence | -0.046 | -0.491-0.399 |
| Magnusson et al. (2012) | STAGE | Emotional neglect | Alcohol dependence | 0.032 | -0.331-0.395 |
| Magnusson et al. (2012) | STAGE | Physical abuse | Alcohol dependence | 0.201 | -0.266-0.669 |
| Magnusson et al. (2012) | STAGE | Sexual abuse | Alcohol dependence | 0.466 | -0.060-0.993 |
| Alemany et al. (2013) | Catalonia twin study | ACEs | Positive psychotic experiences | 0.381 | 0.078-0.685 |
| Alemany et al. (2013) | Catalonia twin study | ACEs | Negative psychotic experiences | 0.390 | 0.086-0.693 |
| Berenz et al. (2013) | NTR | ACEs | Paranoid PD | 0.063 | -0.095-0.221 |
| Berenz et al. (2013) | NTR | ACEs | Schizoid PD | 0.090 | -0.069-0.248 |
| Berenz et al. (2013) | NTR | ACEs | Schizotypal PD | 0.063 | -0.095-0.221 |
| Berenz et al. (2013) | NTR | ACEs | Histrionic PD | 0.063 | -0.095-0.221 |
| Berenz et al. (2013) | NTR | ACEs | Narcissistic PD | 0.127 | -0.031-0.285 |
| Berenz et al. (2013) | NTR | ACEs | Borderline PD | 0.155 | -0.003-0.314 |
| Berenz et al. (2013) | NTR | ACEs | Antisocial PD | 0.155 | -0.003-0.314 |
| Berenz et al. (2013) | NTR | ACEs | Avoidant PD | 0.000 | -0.158-0.158 |
| Berenz et al. (2013) | NTR | ACEs | Obsessive comp. PD | 0.127 | -0.031-0.285 |
| Berenz et al. (2013) | NTR | ACEs | Dependent PD | 0.000 | -0.158-0.158 |
| Bornovalova et al. (2013) | MTFS | Abuse | Borderline PD | 0.090 | -0.086-0.266 |
| Bornovalova et al. (2013) | MTFS | Emotional abuse | Borderline PD | 0.190 | -0.045-0.425 |
| Bornovalova et al. (2013) | MTFS | Physical abuse | Borderline PD | 0.130 | -0.066-0.326 |
| Bornovalova et al. (2013) | MTFS | Sexual abuse | Borderline PD | -0.050 | -0.344-0.244 |
| Voith et al. (2014) | NSCAW-I | ACEs | Trauma | 0.150 | 0.062-0.238 |
| Voith et al. (2014) | NSCAW-I | ACEs | Depression | 0.118 | 0.030-0.206 |
| Barrigon et al. (2015) | Spanish CS study | Maltreatment | Psychosis | 1.096 | 0.033-2.159 |
| Capusan et al. (2016) | STAGE | Maltreatment | ADHD | 0.180 | 0.105-0.255 |
| Capusan et al. (2016) | STAGE | Emotional neglect | ADHD | 0.190 | 0.115-0.265 |
| Capusan et al. (2016) | STAGE | Physical neglect | ADHD | 0.250 | -0.040-0.540 |
| Capusan et al. (2016) | STAGE | Physical abuse | ADHD | 0.080 | -0.065-0.225 |
| Capusan et al. (2016) | STAGE | Sexual abuse | ADHD | 0.200 | 0.020-0.380 |
| Capusan et al. (2016) | STAGE | Maltreatment | ADHD | 0.190 | 0.065-0.315 |
| Capusan et al. (2016) | STAGE | Abuse | ADHD | 0.240 | 0.015-0.465 |
| Capusan et al. (2016) | STAGE | Neglect | ADHD | 0.150 | -0.005-0.305 |
| Dinkler et al. (2017) | CATSS | Maltreatment | ADHD | 0.260 | -0.065-0.585 |
| Dinkler et al. (2017) | CATSS | Maltreatment | Autism | 0.500 | 0.200-0.800 |
| Schaefer et al. (2017) | E-Risk | Victimisation | P-factor | 0.644 | 0.385-0.904 |
| Schaefer et al. (2017) | E-Risk | Victimisation | Internalising factor | 0.655 | 0.449-0.862 |
| Schaefer et al. (2017) | E-Risk | Victimisation | Externalising factor | 0.676 | 0.488-0.863 |
| Schaefer et al. (2017) | E-Risk | Victimisation | Thought disorder | 0.698 | 0.475-0.920 |
| Sonuga-Barke et al. (2017) | ERA | Institutional neglect | ASD (parent, 6y) | 0.772 | 0.340-1.204 |
| Sonuga-Barke et al. (2017) | ERA | Institutional neglect | ASD (parent, 11y) | 1.378 | 0.838-1.919 |
| Sonuga-Barke et al. (2017) | ERA | Institutional neglect | ASD (parent, 15y) | 0.937 | 0.289-1.586 |
| Sonuga-Barke et al. (2017) | ERA | Institutional neglect | ASD (parent, 24.1y) | 0.937 | 0.289-1.586 |
| Sonuga-Barke et al. (2017) | ERA | Institutional neglect | Inattention/overactivity (parent, 6y) | 0.827 | 0.287-1.367 |
| Sonuga-Barke et al. (2017) | ERA | Institutional neglect | Inattention/overactivity (parent, 11y) | 0.551 | 0.011-1.092 |
| Sonuga-Barke et al. (2017) | ERA | Institutional neglect | Inattention/overactivity (parent, 15y) | 0.882 | 0.342-1.422 |
| Sonuga-Barke et al. (2017) | ERA | Institutional neglect | Inattention/overactivity (parent, 24.1y) | 1.048 | 0.399-1.696 |
| Sonuga-Barke et al. (2017) | ERA | Institutional neglect | Emotional problems (parent, 6y) | 0.000 | -0.648-0.648 |
| Sonuga-Barke et al. (2017) | ERA | Institutional neglect | Emotional problems (parent, 11y) | 0.165 | -0.375-0.706 |
| Sonuga-Barke et al. (2017) | ERA | Institutional neglect | Emotional problems (parent, 15y) | 0.551 | -0.205-1.308 |
| Sonuga-Barke et al. (2017) | ERA | Institutional neglect | Emotional problems (parent, 24.1y) | 1.048 | 0.399-1.696 |
| Sonuga-Barke et al. (2017) | ERA | Institutional neglect | Emotional problems (self, 11y) | 0.055 | -0.377-0.487 |
| Sonuga-Barke et al. (2017) | ERA | Institutional neglect | Emotional problems (self, 15y) | 0.221 | -0.212-0.653 |
| Sonuga-Barke et al. (2017) | ERA | Institutional neglect | Emotional problems (self, 24.1y) | 0.717 | 0.176-1.257 |
| Sonuga-Barke et al. (2017) | ERA | Institutional neglect | Conduct problem (parent, 6y) | 0.276 | -0.373-0.924 |
| Sonuga-Barke et al. (2017) | ERA | Institutional neglect | Conduct problem (parent, 11y) | 0.717 | 0.068-1.365 |
| Sonuga-Barke et al. (2017) | ERA | Institutional neglect | Conduct problem (parent, 15y) | 0.276 | -0.373-0.924 |
| Sonuga-Barke et al. (2017) | ERA | Institutional neglect | Conduct problem (parent, 24.1y) | 1.158 | 0.401-1.914 |
| Sonuga-Barke et al. (2017) | ERA | Institutional neglect | Conduct prob (self;11y) | 0.441 | -0.099-0.981 |
| Sonuga-Barke et al. (2017) | ERA | Institutional neglect | Conduct prob (self;15y) | 0.055 | -0.485-0.595 |
| Sonuga-Barke et al. (2017) | ERA | Institutional neglect | Conduct prob (self;24y) | -0.221 | -0.761-0.320 |
| Zvara et al. (2017) | FLP | Sexual abuse | Postnatal depression | 0.290 | 0.016-0.564 |
| Ma et al. (2018) | FFCWS | Physical abuse | Aggressive behaviour | 0.704 | 0.512-0.895 |
| Obikane et al. (2018) | J-SHINE (males) | Physical abuse | Suicidal ideation | 0.532 | 0.298-0.767 |
| Obikane et al. (2018) | J-SHINE (males) | Physical neglect | Suicidal ideation | 0.258 | -0.315-0.832 |
| Obikane et al. (2018) | J-SHINE (males) | Maltreatment | Suicidal ideation | 0.439 | 0.203-0.675 |
| Obikane et al. (2018) | J-SHINE (females) | Physical abuse | Suicidal ideation | 0.576 | 0.363-0.788 |
| Obikane et al. (2018) | J-SHINE (females) | Physical neglect | Suicidal ideation | 0.567 | 0.156-0.978 |
| Obikane et al. (2018) | J-SHINE (females) | Maltreatment | Suicidal ideation | 0.539 | 0.339-0.740 |
| Obikane et al. (2018) | J-SHINE (males) | Physical abuse | Suicidal plan | 0.462 | 0.099-0.826 |
| Obikane et al. (2018) | J-SHINE (males) | Physical neglect | Suicidal plan | 0.167 | -0.437-0.771 |
| Obikane et al. (2018) | J-SHINE (males) | Maltreatment | Suicidal plan | 0.396 | 0.062-0.731 |
| Obikane et al. (2018) | J-SHINE (females) | Physical abuse | Suicidal plan | 0.447 | 0.111-0.782 |
| Obikane et al. (2018) | J-SHINE (females) | Physical neglect | Suicidal plan | 0.314 | -0.170-0.799 |
| Obikane et al. (2018) | J-SHINE (females) | Maltreatment | Suicidal plan | 0.463 | 0.136-0.790 |
| Obikane et al. (2018) | J-SHINE (males) | Physical abuse | Suicide attempt | 0.510 | 0.060-0.961 |
| Obikane et al. (2018) | J-SHINE (males) | Physical neglect | Suicide attempt | -0.348 | -1.323-0.626 |
| Obikane et al. (2018) | J-SHINE (males) | Maltreatment | Suicide attempt | 0.358 | -0.127-0.842 |
| Obikane et al. (2018) | J-SHINE (females) | Physical abuse | Suicide attempt | 0.651 | 0.378-0.924 |
| Obikane et al. (2018) | J-SHINE (females) | Physical neglect | Suicide attempt | 0.961 | 0.400-1.521 |
| Obikane et al. (2018) | J-SHINE (females) | Maltreatment | Suicide attempt | 0.659 | 0.394-0.924 |
| Stern et al. (2018) | E-Risk | Victimisation (5-12y) | ADHD (5-12y) | 0.140 | 0.022-0.259 |
| Stern et al. (2018) | E-Risk | Victimisation (18y) | ADHD (18y) | 0.345 | 0.225-0.465 |
| Baldwin et al. (2019) | E-Risk | Victimisation | Suicidal ideation | 0.205 | 0.053-0.357 |
| Baldwin et al. (2019) | E-Risk | Victimisation | Self harm | 0.224 | 0.091-0.356 |
| Baldwin et al. (2019) | E-Risk | Victimisation | Suicide attempt | 0.136 | -0.104-0.376 |
| Gerin et al. (2019) | DNS | Maltreatment | Internalising | 0.620 | 0.326-0.914 |
| Kugler et al. (2019) | FADS | Maltreatment | Drug use | 0.362 | 0.156-0.568 |
| Kugler et al. (2019) | FADS | Maltreatment | Depression | 0.235 | 0.030-0.441 |
| Lecei et al. (2019) | TwinssCan | Maltreatment | Total psychopathology | 0.295 | 0.054-0.537 |
| Lecei et al. (2019) | TwinssCan | Maltreatment | Psychosis | 0.270 | 0.029-0.512 |
| Lecei et al. (2019) | TwinssCan | Maltreatment | Anxiety | 0.386 | 0.143-0.628 |
| Lecei et al. (2019) | TwinssCan | Maltreatment | Depression | 0.254 | 0.012-0.495 |
| Schwartz et al. (2019) | MIDUS | ACEs | Depression | 0.116 | -0.018-0.249 |
| Schwartz et al. (2019) | MIDUS | ACEs | Antisocial behaviour | 0.226 | 0.093-0.360 |
| Schwartz et al. (2019) | Add Health | ACEs | Depression | 0.107 | 0.036-0.177 |
| Alvanzo et al. (2020) | NESARC (males) | ACEs | Severe alcohol problems | -0.090 | -0.329-0.150 |
| Alvanzo et al. (2020) | NESARC (females) | ACEs | Severe alcohol problems | 0.005 | -0.222-0.233 |
| Alvanzo et al. (2020) | NESARC (males) | ACEs | Moderate alcohol problems | 0.067 | -0.106-0.241 |
| Alvanzo et al. (2020) | NESARC (females) | ACEs | Moderate alcohol problems | 0.136 | -0.028-0.300 |
| Golm et al. (2020) | ERA | Institutional neglect | Depression (parent) | 0.480 | 0.088-0.872 |
| Golm et al. (2020) | ERA | Institutional neglect | Generalised anxiety (parent) | 0.490 | 0.098-0.882 |
| Golm et al. (2020) | ERA | Institutional neglect | Depression (self) | 0.410 | -0.002-0.822 |
| Golm et al. (2020) | ERA | Institutional neglect | Generalised anxiety (self) | 0.380 | -0.032-0.792 |
| Kullberg et al. (2020) | NESDA | Emotional abuse | Depression | 0.509 | 0.351-0.667 |
| Kullberg et al. (2020) | NESDA | Physical abuse | Depression | -0.002 | -0.158-0.153 |
| Kullberg et al. (2020) | NESDA | Sexual abuse | Depression | 0.068 | -0.087-0.224 |
| Kullberg et al. (2020) | NESDA | Emotional abuse | Anxiety | 0.292 | 0.135-0.448 |
| Kullberg et al. (2020) | NESDA | Physical abuse | Anxiety | 0.073 | -0.083-0.228 |
| Kullberg et al. (2020) | NESDA | Sexual abuse | Anxiety | 0.104 | -0.051-0.260 |
| Capusan et al. (2021) | Östergötland cohort | Maltreatment | Substance use disorder | 0.771 | 0.488-1.053 |
| Isumi et al. (2021) | A-CHILD | Maltreatment | Behavioural difficulties | 0.333 | 0.281-0.384 |
| Li et al. (2021) | Chinese long. study | Emotional abuse | Depression | 0.100 | 0.037-0.163 |
| Li et al. (2021) | Chinese long. study | Emotional abuse | Depression | 0.100 | 0.037-0.163 |
| Li et al. (2021) | Chinese long. study | Emotional abuse | Depression | 0.100 | 0.037-0.163 |
| Li et al. (2021) | Chinese long. study | Emotional abuse | Depression | 0.080 | 0.017-0.143 |

## Table S10. Meta-analytic effect sizes for the relationship between maltreatment and mental health outcomes reported in previous meta-analyses of non-quasi-experimental studies.

| **Reference** | **Outcome** | **Maltreatment type** | **Odds Ratio** | **Cohen's D** |
| --- | --- | --- | --- | --- |
| Nelson et al. (2017) | Depression | Sexual abuse | 2.66 (2.38-2.98) | 0.54 (0.03) |
| Nelson et al. (2017) | Depression | Physical abuse | 2.68 (2.29-3.12) | 0.54 (0.04) |
| Nelson et al. (2017) | Depression | Emotional abuse | 3.73 (2.88-4.83) | 0.73 (0.07) |
| Nelson et al. (2017) | Depression | Emotional neglect | 3.54 (2.48-5.04) | 0.70 (0.10) |
| Nelson et al. (2017) | Depression | Physical neglect | 2.45 (1.63-3.68) | 0.49 (0.11) |
| Nelson et al. (2017) | Depression | Any | 2.81 (2.35-3.36) | 0.57 (0.05) |
| Li et al. (2016) | Depression | Any | 2.03 (1.37-3.01) | 0.39 (0.11) |
| Li et al. (2016) | Anxiety | Any | 2.70 (2.10-3.47) | 0.55 (0.07) |
| Varese et al. (2012) | Psychosis | Any | 2.78 (2.34-3.31) | 0.56 (0.05) |
| Liu et al. (2018) | NSSI | Any | 3.42 (2.74-4.26) | 0.68 (0.06) |
| Liu et al. (2018) | NSSI | Sexual abuse | 2.65 (2.33-3.03) | 0.54 (0.04) |
| Liu et al. (2018) | NSSI | Physical abuse | 2.31 (1.97-2.69) | 0.46 (0.04) |
| Liu et al. (2018) | NSSI | Physical neglect | 2.22 (1.75-2.80) | 0.44 (0.07) |
| Liu et al. (2018) | NSSI | Emotional abuse | 3.03 (2.59-3.54) | 0.61 (0.04) |
| Liu et al. (2018) | NSSI | Emotional neglect | 1.84 (1.45-2.34) | 0.34 (0.07) |
| Angelaskis et al. (2019) | Suicide attempt | Sexual abuse | 3.17 (2.76-3.64) | 0.64 (0.04) |
| Angelaskis et al. (2019) | Suicide attempt | Physical abuse | 2.52 (2.09-3.04) | 0.51 (0.05) |
| Angelaskis et al. (2019) | Suicide attempt | Emotional abuse | 2.49 (1.64-3.77) | 0.50 (0.12) |
| Angelaskis et al. (2019) | Suicide attempt | Any | 2.09 (1.67-2.60) | 0.41 (0.06) |
| Angelaskis et al. (2019) | Suicide attempt | Emotional neglect | 2.29 (1.79-2.94) | 0.46 (0.07) |
| Angelaskis et al. (2019) | Suicide attempt | Physical neglect | 1.51 (0.87-2.61) | 0.23 (0.15) |
| Angelaskis et al. (2019) | Suicide attempt | Complex abuse | 5.18 (2.52-1.63) | 0.91 (0.06) |
| Angelaskis et al. (2019) | Suicidal ideation | Sexual abuse | 2.15 (1.77-2.61) | 0.42 (0.05) |
| Angelaskis et al. (2019) | Suicidal ideation | Physical abuse | 2.43 (1.85-3.18) | 0.49 (0.08) |
| Angelaskis et al. (2019) | Suicidal ideation | Emotional abuse | 2.10 (1.51-2.94) | 0.41 (0.09) |
| Angelaskis et al. (2019) | Suicidal ideation | Any | 2.66 (1.93-3.68) | 0.54 (0.09) |
| Angelaskis et al. (2019) | Suicidal ideation | Emotional neglect | 1.40 (1.02-1.93) | 0.19 (0.09) |
| Angelaskis et al. (2019) | Suicidal ideation | Physical neglect | 1.44 (1.06-1.95) | 0.20 (0.09) |
| Halpern et al. (2018) | Substance abuse | Physical abuse | 1.74 (1.36-2.21) | 0.31 (0.07) |
| Halpern et al. (2018) | Substance abuse | Sexual abuse | 1.73 (1.24-2.41) | 0.30 (0.09) |
| Halpern et al. (2018) | Substance abuse | Neglect | 1.19 (0.92-1.52) | 0.10 (0.07) |
| Braga et al. (2018) | Antisocial behaviour | Any | 1.96 (1.42-2.71) | 0.37 (0.09) |
| Hailes et al. (2019) | Schizophrenia | Sexual abuse | 1.40 (0.80-2.30) | 0.19 (0.15) |
| Hailes et al. (2019) | Eating disorders | Sexual abuse | 2.20 (1.80-2.80) | 0.43 (0.06) |
| Hailes et al. (2019) | PTSD | Sexual abuse | 2.30 (1.60-3.40) | 0.46 (0.11) |
| Hailes et al. (2019) | Depression | Sexual abuse | 2.70 (2.40-3.00) | 0.55 (0.03) |
| Hailes et al. (2019) | Anxiety | Sexual abuse | 2.70 (2.50-2.80) | 0.55 (0.02) |
| Hailes et al. (2019) | Borderline personality disorder | Sexual abuse | 2.90 (2.50-3.30) | 0.59 (0.04) |
| Pooled effect size |  |  | 2.36 (2.07-2.7) | 0.48 (0.04) |

Note. We selected the most recent meta-analyses that assessed the relationships between child maltreatment and the mental health outcomes examined in this meta-analysis. Results were extracted as odds ratios from original meta-analyses and were converted to Cohen’s d values for comparability with effect sizes presented in this meta-analysis.

## Figure S1. Study selection procedure

**Identification of studies via databases**

Records removed *before screening*:

Duplicate records removed (n=3,428)

Records identified from databases (n=9,689)

**Identification**

Records screened

(n=6,261)

Records excluded

(n=6,142)

Reports sought for retrieval

(n=119)

Reports not retrieved

(n=0)

**Screening**

Reports excluded:

Wrong study design (n=45)

Wrong outcome (n=15)

Wrong exposure (n=13)

Duplicate results from overlapping sample (n=8)

Conference abstract (n=3)

Effect size could not be converted (n=1)*

Reports assessed for eligibility

(n=119)

Studies included in quantitative synthesis (meta-analysis)

(n=34)

**Included**

Note: *A Mendelian Randomisation study ^32^ met criteria for inclusion, but the effect sizes could not be accurately converted to Cohen’s d (following consultation with the authors), so it was not included in the meta-analysis.

## Figure S2. Meta-analysis of the unadjusted association between childhood maltreatment and mental health problems

Note. $I^{2}$ for the MREM was 97.29, indicating that 97% of variation between effect sizes would remain if sampling error was eliminated.

## Figure S3. Funnel plots

A shows all quasi-experimental adjusted effect sizes; B shows quasi-experimental adjusted effect sizes excluding the ERA Study (shown in pink in A). Colours represent independent samples.

## Figure S**4**. P-curve analysis across study-averaged effect sizes

Note. The shape of the distribution of p-values diagnoses whether the findings contain evidential value, or whether selective reporting of studies (i.e., file-drawering) or analyses (i.e., p-hacking) are the only cause of statistically significant findings. True findings produce p-curves with a right-skewed distribution, containing more low than high statistically significant p-values (e.g., p$\leq$0.01 vs p~0.04). Therefore, statistically significant right-skewed p-curves (tested against a null of no effect; see red dotted line) reflect evidential value. In contrast, findings linked to p-hacking or selective reporting produce p-curves with flat or left skew, with at least as many high significant p-values (e.g., p~0.04) than low (p$\leq$0.01). To test whether the p-curve is flat, the observed p-curve is compared against a p-curve that would be expected if the studies had 33% power (see green dashed line), given that studies with greater power yield a steeper right-skewed p-curve.^33^ If the observed p-curve is significantly flatter than the null of 33% power, it suggests that the p-curve is flat. This p-curve includes 27 statistically significant (p<0.05) results, of which 23 are p<0.025. There were 7 additional results excluded from p-curve analysis because they were p>0.05.

## Figure S**5**. Leave-one-out analysis in which each cohort was omitted in turn from the meta-analysis

Note. Results were also consistent when removing each of the 156 effect sizes in turn: the meta-analytic effect ranged from ranged between Cohen’s d = 0.29 to 0.31.

## Figure S6. Leave-one-out analysis in which each cohort was omitted in turn from the meta-analysis

Note. Results were also consistent when removing each of the 156 effect sizes in turn: the meta-analytic effect ranged from ranged between Cohen’s d = 0.29 to 0.31.

References

1. Rutter M, Sonuga‐Barke EJ, Castle J. I. Investigating the impact of early institutional deprivation on development: Background and research strategy of the English and Romanian Adoptees (ERA) study. *Monogr Soc Res Child Dev*. 2010;75(1):1-20.

2. Nelson EC, Heath AC, Lynskey MT, et al. Childhood sexual abuse and risks for licit and illicit drug-related outcomes: a twin study. *Psychol Med*. 2006;36(10):1473-1483.

3. Kendler KS, Bulik CM, Silberg J, Hettema JM, Myers J, Prescott CAJAogp. Childhood sexual abuse and adult psychiatric and substance use disorders in women: an epidemiological and cotwin control analysis. *JAMA Psychiatry*. 2000;57(10):953-959.

4. Beckett C, Bredenkamp D, Castle J, Groothues C, O’connor TG, Rutter M. Behavior patterns associated with institutional deprivation: A study of children adopted from Romania. *J Dev Behav Pediatr*. 2002;23(5):297-303.

5. Schaefer JD, Moffitt TE, Arseneault L, et al. Adolescent victimization and early-adult psychopathology: Approaching causal inference using a longitudinal twin study to rule out non-causal explanations. *Clin Psychol Sci*. 2017;6(3):352–371. doi:doi.org/10.1177/2167702617741381

6. Baldwin JR, Arseneault A, Caspi A, et al. Adolescent victimization and self-injurious thoughts and behaviors: A genetically sensitive cohort study. *J Am Acad Child Adolesc Psychiatry*. 2019;58(5):506-513.

7. Dinkler L, Lundström S, Gajwani R, Lichtenstein P, Gillberg C, Minnis H. Maltreatment‐associated neurodevelopmental disorders: a co‐twin control analysis. *J Child Psychol Psychiatry*. 2017;58(6):691-701.

8. Gerin MI, Viding E, Pingault JB, et al. Heightened amygdala reactivity and increased stress generation predict internalizing symptoms in adults following childhood maltreatment. *J Child Psychol Psychiatry*. 2019;60(7):752-761.

9. Capusan AJ, Kuja-Halkola R, Bendtsen P, et al. Childhood maltreatment and attention deficit hyperactivity disorder symptoms in adults: a large twin study. *Psychol Med*. 2016;46(12):2637-2646.

10. Magnusson Å, Lundholm C, Göransson M, Copeland W, Heilig M, Pedersen N. Familial influence and childhood trauma in female alcoholism. *Psychol Med*. 2012;42(2):381-389.

11. Young-Wolff KC, Kendler K, Ericson M, Prescott C. Accounting for the association between childhood maltreatment and alcohol-use disorders in males: a twin study. *Psychol Med*. 2011;41(1):59-70.

12. Nelson EC, Heath AC, Madden PA, et al. Association between self-reported childhood sexual abuse and adverse psychosocial outcomes: results from a twin study. *Arch Gen Psychiatry*. 2002;59(2):139-145.

13. Dinwiddie S, Heath AC, Dunne MP, et al. Early sexual abuse and lifetime psychopathology: a co-twin–control study. *Psychol Med*. 2000;30(1):41-52.

14. Schwartz JA, Wright EM, Valgardson BA. Adverse childhood experiences and deleterious outcomes in adulthood: A consideration of the simultaneous role of genetic and environmental influences in two independent samples from the United States. *Child Abuse Neglect*. 2019;88:420-431.

15. Obikane E, Shinozaki T, Takagi D, Kawakami N. Impact of childhood abuse on suicide-related behavior: analysis using marginal structural models. *J Affect Disord*. 2018;234:224-230.

16. Barrigón ML, Diaz FJ, Gurpegui M, et al. Childhood trauma as a risk factor for psychosis: A sib-pair study. *J Psychiatr Res*. 2015;70:130-136.

17. Alvanzo AA, Storr CL, Reboussin B, et al. Adverse childhood experiences (ACEs) and transitions in stages of alcohol involvement among US adults: Progression and regression. *Child Abuse Neglect*. 2020;107:104624.

18. Cochrane. Obtaining standard errors from confidence intervals and P values: absolute (difference) measures. Accessed 13 September 2021, <https://handbook-5-1.cochrane.org/chapter_7/7_7_7_2_obtaining_standard_errors_from_confidence_intervals_and.htm>

19. Stern A, Agnew-Blais J, Danese A, et al. Associations between abuse/neglect and ADHD from childhood to young adulthood: a prospective nationally-representative twin study. *Child Abuse Neglect*. 2018;81:274-285.

20. Riggins-Caspers KM, Cadoret RJ, Knutson JF, Langbehn D. Biology-environment interaction and evocative biology-environment correlation: Contributions of harsh discipline and parental psychopathology to problem adolescent behaviors. *Behav Genet*. 2003;33(3):205-220.

21. Voith LA, Gromoske AN, Holmes MR. Effects of cumulative violence exposure on children’s trauma and depression symptoms: A social ecological examination using fixed effects regression. *J Child Adolesc Trauma*. 2014;7(4):207-216.

22. Berenz EC, Amstadter AB, Aggen SH, et al. Childhood trauma and personality disorder criterion counts: a co-twin control analysis. *Journal of abnormal psychology*. 2013;122(4):1070.

23. Altman DG, Bland JM. How to obtain the confidence interval from a P value. *BMJ*. 2011;343

24. Jaffee SR, Caspi A, Moffitt TE, Taylor A. Physical maltreatment victim to antisocial child: evidence of an environmentally mediated process. *Journal of Abnormal Psychology*. 2004;113(1):44-55.

25. Sonuga-Barke EJ, Kennedy M, Kumsta R, et al. Child-to-adult neurodevelopmental and mental health trajectories after early life deprivation: the young adult follow-up of the longitudinal English and Romanian Adoptees study. *The Lancet*. 2017;389(10078):1539-1548.

26. Rutter M. Developmental catch-up, and deficit, following adoption after severe global early privation. *The Journal of Child Psychology and Psychiatry and Allied Disciplines*. 1998;39(4):465-476.

27. Borenstein M, Hedges LV, Higgins JP, Rothstein HR. Effect sizes based on means. *Introduction to meta-analysis*. 2009:21-32.

28. Borenstein M, Hedges LV, Higgins JP, Rothstein HR. Converting among effect sizes. *Introduction to meta-analysis*. 2009:45-49.

29. Lipsey MW, Wilson DB. *Practical meta-analysis*. SAGE publications, Inc; 2001.

30. Grant RL. Converting an odds ratio to a range of plausible relative risks for better communication of research findings. *BMJ*. 2014;348

31. Capusan AJ, Gustafsson PA, Kuja-Halkola R, Igelström K, Mayo LM, Heilig M. Re-examining the link between childhood maltreatment and substance use disorder: a prospective, genetically informative study. *Mol Psychiatry*. 2021:1-9.

32. Warrier V, Kwong AS, Luo M, et al. Gene–environment correlations and causal effects of childhood maltreatment on physical and mental health: a genetically informed approach. *Lancet Psychiatry*. 2021;8(5):373-386.

33. Simonsohn U, Nelson LD, Simmons JP. P-curve: a key to the file-drawer. *J Exp Psychol Gen*. 2014;143(2):534.
